# Characterization of binding, functional activity, and contractile responses of the selective 5‐HT_1F_ receptor agonist lasmiditan

**DOI:** 10.1111/bph.14832

**Published:** 2019-11-07

**Authors:** Eloísa Rubio‐Beltrán, Alejandro Labastida‐Ramírez, Kristian A. Haanes, Antoon van den Bogaerdt, Ad J.J.C. Bogers, Eric Zanelli, Laurent Meeus, A.H. Jan Danser, Michael R. Gralinski, Peter B. Senese, Kirk W. Johnson, Joseph Kovalchin, Carlos M. Villalón, Antoinette MaassenVanDenBrink

**Affiliations:** ^1^ Division of Pharmacology, Department of Internal Medicine Erasmus University Medical Centre Rotterdam The Netherlands; ^2^ Department of Cardiothoracic Surgery Erasmus University Medical Centre Rotterdam The Netherlands; ^3^ Research and Development Déclion Pharmaceuticals, Inc. Marblehead Massachusetts; ^4^ Euroscreen Fast Services Unit Epics Therapeutics SA Gosselies Belgium; ^5^ CorDynamics, Inc. Chicago Illinois; ^6^ Lilly Corporate Center Eli Lilly and Company Indianapolis Indiana; ^7^ Research and Development CoLucid Pharmaceuticals, Inc. Cambridge Massachusetts; ^8^ Pharmacobiology Cinvestav‐Coapa Mexico City Mexico

## Abstract

**Background and Purpose:**

Triptans are 5‐HT_1B/1D_ receptor agonists (that also display 5‐HT_1F_ receptor affinity) with antimigraine action, contraindicated in patients with coronary artery disease due to their vasoconstrictor properties. Conversely, lasmiditan was developed as an antimigraine 5‐HT_1F_ receptor agonist. To assess the selectivity and cardiovascular effects of lasmiditan, we investigated the binding, functional activity, and *in vitro/in vivo* vascular effects of lasmiditan and compared it to sumatriptan.

**Experimental Approach:**

Binding and second messenger activity assays of lasmiditan and other serotoninergic agonists were performed for human 5‐HT_1A_, 5‐HT_1B_, 5‐HT_1D_, 5‐ht_1E_, 5‐HT_1F_, 5‐HT_2A_, 5‐HT_2B_, and 5‐HT_7_ receptors, and the results were correlated with their potency to constrict isolated human coronary arteries (HCAs). Furthermore, concentration–response curves to lasmiditan and sumatriptan were performed in proximal and distal HCA, internal mammary, and middle meningeal arteries. Finally, anaesthetized female beagle dogs received i.v. infusions of lasmiditan or sumatriptan in escalating cumulative doses, and carotid and coronary artery diameters were measured.

**Key Results:**

Lasmiditan showed high selectivity for 5‐HT_1F_ receptors. Moreover, the functional potency of the analysed compounds to inhibit cAMP increase through 5‐HT_1B_ receptor activation positively correlated with their potency to contract HCA. In isolated human arteries, sumatriptan, but not lasmiditan, induced contractions. Likewise, *in vivo*, sumatriptan decreased coronary and carotid artery diameters at clinically relevant doses, while lasmiditan was devoid of vasoconstrictor activity at all doses tested.

**Conclusions and Implications:**

Lasmiditan is a selective 5‐HT_1F_ receptor agonist devoid of vasoconstrictor activity. This may represent a cardiovascular safety advantage when compared to the triptans.

**What is already known**

- Lasmiditan is effective in the acute treatment of migraine.

**What this study adds**

- Lasmiditan selectively activates the human 5‐HT_1F_ receptor and is devoid of vasoconstrictor activity.

**What is the clinical significance**

- The lack of vasoconstriction may represent a safety advantage for migraine patients with cardiovascular disease.

**Abbreviations**
5‐CT: 5‐carboxamidotryptamine
ANCOVA: analysis of covariance
DAP: diastolic arterial pressure
HCA: human coronary artery
LCX: left circumflex
MAP: mean arterial pressure
RANCOVA: repeated measure analysis of covariance
SAP: systolic arterial pressure

## 1 INTRODUCTION

Migraine is a neurological disease characterized by throbbing unilateral headaches of moderate to severe intensity, accompanied by nausea, vomiting, photophobia, and/or phonophobia (Headache Classification Committee of the International Headache Society, 2018). It has an estimated prevalence of 15% in the global population, with women being three times more affected than men (Kurth et al., 2016; Vos et al., 2012).

Currently, the specific therapies for acute antimigraine treatment are the triptans, selective https://www.guidetopharmacology.org/GRAC/ObjectDisplayForward?objectId=2616_/_ https://www.guidetopharmacology.org/GRAC/ObjectDisplayForward?objectId=3 receptor agonists that also display varying levels of https://www.guidetopharmacology.org/GRAC/ObjectDisplayForward?objectId=5_1F_ receptor affinity. Unfortunately, not all patients respond to triptans (Diener & Limmroth, 2001), and they are contraindicated in patients with cardiovascular disease, due to their contractile properties via activation of 5‐HT_1B_ receptors in coronary arteries (Chan et al., 2014; Dodick et al., 2004; Labruijere et al., 2015; MaassenVanDenBrink, Reekers, Bax, Ferrari, & Saxena, 1998). Therefore, there is a need for novel antimigraine drugs for the patients that do not respond to the current available treatments, but also, for those patients with cardiovascular disease.

Based on results from preclinical studies, the 5‐HT_1F_ receptor agonist, https://www.guidetopharmacology.org/GRAC/LigandDisplayForward?ligandId=3928, was developed for acute antimigraine treatment (Johnson et al., 1997; Nelson et al., 2010) and Phase III trials showed positive results (Goadsby et al., 2019; Kuca, Silberstein, Wietecha, Berg, Dozier, & Lipton, 2018). Considering the increased cardiovascular risk of migraine patients (Buse, Reed, Fanning, Kurth, & Lipton, 2017; Kurth et al., 2016; Sacco & Kurth, 2014; Schurks et al., 2009), and the presence of 5‐HT_1F_ receptors in the vasculature (Bouchelet, Case, Olivier, & Hamel, 2000; Bouchelet, Cohen, Case, Séguéla, & Hamel, 1996; Nilsson et al., 1999), it is important to determine whether lasmiditan lacks affinity for *human* 5‐HT_1B_ receptors and whether activation of 5‐HT_1F_ receptors will result in vasoconstrictive responses. On this basis, the aim of this study was to investigate the pharmacological properties of lasmiditan and in particular (a) to assess the selectivity and functional activity of lasmiditan, triptans, and other 5‐HT receptor ligands on various *human* 5‐HT receptors; (b) to analyse its potential to induce vasoconstriction in *in vitro* (isolated human proximal and distal coronary, internal mammary, and middle meningeal arteries) and in vivo (carotid and coronary artery diameters in anesthetized dogs) preclinical models; and (c) to compare our findings with lasmiditan to those obtained with https://www.guidetopharmacology.org/GRAC/LigandDisplayForward?ligandId=54, one of the most prescribed triptans to acutely treat migraine.

We hypothesize that, unlike sumatriptan, lasmiditan selectively activates the *human* 5‐HT_1F_ receptor and does not induce vasoconstriction in the above *in vitro* (including human coronary arteries) and *in vivo* vascular models.

## 2 MATERIALS AND METHODS

### 2.1 Cell membrane preparation

CHO‐K1 cells (RRID:CVCL_0214) expressing the human recombinant https://www.guidetopharmacology.org/GRAC/ObjectDisplayForward?objectId=1_1A_, 5‐HT_1B_, https://www.guidetopharmacology.org/GRAC/ObjectDisplayForward?objectId=3_1D_, https://www.guidetopharmacology.org/GRAC/ObjectDisplayForward?objectId=4_1E_, 5‐HT_1F_, https://www.guidetopharmacology.org/GRAC/ObjectDisplayForward?objectId=6_2A_, https://www.guidetopharmacology.org/GRAC/ObjectDisplayForward?objectId=7_2B_, or https://www.guidetopharmacology.org/GRAC/ObjectDisplayForward?objectId=12_7_ receptors were grown prior to the test in media without antibiotic at Ogeda SA (Gosselies, Belgium). Cells were prepared using a protocol from Ogeda. In brief, cells were harvested by scraping from the culture vessels in ice‐cold Ca^2+^‐ and Mg^2+^‐free PBS. The cells were then centrifuged for 10 min at 5,000×*g* and 4°C, and the pellets were suspended in buffer A (15‐mM Tris–HCl pH 7.5, 2‐mM MgCl_2_, 0.3‐mM EDTA, and 1‐mM EGTA) and homogenized in a glass–glass homogenizer. The crude membrane fraction was collected by two consecutive centrifugation steps at 35,000×*g* and 4°C for 30 min separated by a washing step in buffer A. The final membrane pellet was suspended in buffer B (75‐mM Tris–HCl pH 7.5, 12.5‐mM MgCl_2_, 0.3‐mM EDTA, 1‐mM EGTA, and 250‐mM sucrose) and flash‐frozen in liquid nitrogen. Protein content was determined by the BCA method (Interchim, UP40840A).

### 2.2 Radioligand binding competition assay

Competition binding was performed in duplicate in the wells of a 96‐well plate containing binding buffer (optimized for each receptor), cell membrane extracts (approximately 20,000 cells distributed in the 96‐well plate), radiotracer, and test agonist. Non‐specific binding was determined by co‐incubation with 200‐fold excess of competitor. Cells were incubated and exposed to varying concentrations (1 pM to 10 μM) of a range of displacer agonists (see below). The samples were incubated in a final volume of 0.1 ml and then filtered over Unifilter plates (Perkin Elmer, Massachusetts, USA) pretreated for 2 hr to limit tracer non‐specific binding. Filters were washed five times with 0.5 ml of ice‐cold washing buffer (tris 50 mM pH 7.4) and 50 μl of Microscint 20 (Perkin Elmer) were added to each filter. The plates were incubated 15 min at room temperature on an orbital shaker and then counted with a TopCount™ (Perkin Elmer) for 1 min per well.

#### 2.2.1 cAMP HTRF assay for G_i_ coupled receptors

Concentration–response curves were performed in parallel with the agonists. For agonist tests, 12 μl of cells were mixed with 6 μl of the test compound (at increasing concentrations) and 6 μl of https://www.guidetopharmacology.org/GRAC/LigandDisplayForward?ligandId=5190 and then incubated for 30 min at room temperature. After addition of the lysis buffer and 1‐hr incubation, cAMP concentrations were estimated according to the manufacturer specification with the HTRF kit (Cisbio International, Codelet, France). In brief, increasing concentrations of agonists were added to stably transfected cells in buffer in an Optiplate (PerkinElmer Life Sciences, Massachusetts, USA). The plates were incubated, and cells were then lysed by the addition of HTRF reagents (cAMP‐Cryptate and anti‐cAMP‐d_2_ reagents) and diluted in lysis buffer, followed by incubation at room temperature. As 5‐HT_1B_ receptors have been reported in naïve CHO‐K1 cells (George, Bungay, & Naylor, 1997), we also tested https://www.guidetopharmacology.org/GRAC/LigandDisplayForward?ligandId=4 (5‐CT, the reference agonist for the 5‐HT_1B_ receptor), in CHO‐K1 cells transfected with a non‐5‐HT, GPCR.

#### 2.2.2 cAMP HTRF assay for G_s_ coupled receptors

Concentration–response curves were performed in parallel. For agonist tests, 12 μl of cells were mixed with 12 μl of the test compound at increasing concentrations and then incubated 30 min at room temperature. After addition of the lysis buffer and 60 min incubation, cAMP concentrations were estimated, according to the manufacturer specification, with the HTRF kit (Cisbio International, Codelet, France). Briefly, increasing concentrations of agonists were added to stably transfected cells in buffer in an Optiplate (PerkinElmer Life Sciences). The plates were incubated, and cells were then lysed by the addition of HTRF reagents (cAMP‐Cryptate and anti‐cAMP‐d_2_ reagents) and diluted in lysis buffer, followed by incubation at room temperature.

#### 2.2.3 IPOne HTRF assay

The assay was performed on adherent cells. For agonist testing, the medium was removed and 20 μl of assay buffer plus 20 μl of the studied agonist or the reference agonist were added in each well. The plate was incubated for 60 min at 37°C with 5% CO_2_. IP_1_‐D2 reagent and anti‐IP_1_ cryptate reagents were dispensed in the wells, and IP_1_ concentrations were then measured following the manufacturer instructions (IPOne HTRF assay kit; Cisbio International, Codolet, France). In brief, increasing concentrations of agonists were added to stably transfected cells in buffer in an Optiplate (PerkinElmer Life Sciences). The plates were incubated, and cells were then lysed by the addition of HTRF reagents (IP_1_‐D2 reagent and anti‐IP_1_ cryptate reagents) and diluted in lysis buffer, followed by incubation at room temperature.

#### 2.2.4 GTP_γ_^35^S assay

For agonist testing, membrane extracts expressing the receptor of interest was mixed with GDP. In parallel, GTP_γ_[^35^S] was mixed with the beads just before starting the reaction. The following reagents were successively added in the wells of an Optiplate (Perkin Elmer): 50 μl of reference ligand, 10 μl of assay buffer, 20 μl of the cells: GDP mix, and 20 μl of the GTP_γ_[^35^S]: beads mix. The plate was incubated for 60 min, then centrifuged and counted with a PerkinElmer TopCount™ reader.

#### 2.2.5 Agonists tested

5‐HT (shttps://www.guidetopharmacology.org/GRAC/LigandDisplayForward?ligandId=5190), 5‐CT, https://www.guidetopharmacology.org/GRAC/LigandDisplayForward?ligandId=149, sumatriptan, https://www.guidetopharmacology.org/GRAC/LigandDisplayForward?ligandId=60, https://www.guidetopharmacology.org/GRAC/LigandDisplayForward?ligandId=45, https://www.guidetopharmacology.org/GRAC/LigandDisplayForward?ligandId=5190, https://www.guidetopharmacology.org/GRAC/LigandDisplayForward?ligandId=7110, https://www.guidetopharmacology.org/GRAC/LigandDisplayForward?ligandId=40, https://www.guidetopharmacology.org/GRAC/LigandDisplayForward?ligandId=7191, https://www.guidetopharmacology.org/GRAC/LigandDisplayForward?ligandId=3928, avitriptan, https://www.guidetopharmacology.org/GRAC/LigandDisplayForward?ligandId=120, lasmiditan, https://www.guidetopharmacology.org/GRAC/LigandDisplayForward?ligandId=20, and https://www.guidetopharmacology.org/GRAC/LigandDisplayForward?ligandId=21 were tested. The radioligands and reference compounds used for the radioligand and second messenger studies are specified in Tables S1 and S2.

### 2.3 Human isolated arteries

#### 2.3.1 Coronary arteries

Coronary arteries were obtained from six “heart beating” organ donors (three males and three females; 48–62 years), who died of non‐cardiac disorders less than 24 hr before the tissue was taken to the laboratory. The hearts were provided by the Heart Valve Bank Beverwijk Bank (nowadays ETB‐BISLIFE Tissue Bank) at that time still located in Rotterdam, from Dutch post‐mortem donors, after donor mediation via Bio Implant Services/Eurotransplant Foundation (Leiden, The Netherlands), following removal of the aortic and pulmonary valves for homograft valve transplantation. All donors gave permission for research. Immediately after circulatory arrest, the hearts were stored at 4°C in a sterile organ protecting solution and were brought to the laboratory within the first 24 hr of death. After arrival at the laboratory, the right proximal (internal diameter 3–5 mm) and distal (internal diameter 0.5–1 mm) portions of the coronary artery were dissected and placed in a cold, oxygenated (95% O_2_/5% CO_2_) Krebs buffer solution of the following composition: 118‐mM NaCl, 4.7‐mM KCl, 2.5‐mM CaCl_2_, 1.2‐mM MgSO_4_, 1.2‐mM KH_2_PO_4_, 25‐mM NaHCO_3_, and 8.3‐mM glucose; pH 7.4.

#### 2.3.2 Internal mammary arteries

Internal mammary arteries (internal diameter 2–3 mm) were obtained perioperatively from 18 patients (16 males and two females; 51–80 years) undergoing coronary bypass surgery. The tissue was immediately placed in a sterile organ‐protecting solution and was brought to the laboratory within 15 min. Subsequently, the artery was cleaned of connective tissue and placed in a cold, oxygenated Krebs buffer solution (for composition, see above).

#### 2.3.3 Middle meningeal arteries

Middle meningeal arteries (internal diameter 0.5–1.5 mm) were obtained from the dura mater of six patients (two males and four females; 12–68 years) who underwent neurosurgery. The dura mater, together with a small piece of the meningeal artery, was collected in a sterile organ‐protecting solution and immediately transported to the laboratory. The dura mater and connective tissue were dissected, and the artery was placed in a cold, oxygenated Krebs solution of the following composition: 119‐mM NaCl, 4.7‐mM KCl, 1.25‐mM CaCl_2_, 1.2‐mM MgSO_4_, 1.2‐mM KH_2_PO_4_, 25‐mM NaHCO_2_, and 11.1‐mM glucose; pH 7.4.

All arteries were used on the same day or stored overnight and used the following day for functional experiments. The studies on coronary arteries were approved by the Scientific Advisory Board of the Rotterdam Heart Valve Bank. The Medical Ethics Committee of the Erasmus Medical Center, Rotterdam, approved the study protocols with regard to mammary arteries and middle meningeal arteries.

### 2.4 Isometric tension measurements

Proximal coronary arteries were cut into segments of 2‐ to 4‐mm length, excluding distinct, macroscopically visible atherosclerotic lesions. The segments were mounted on stainless steel hooks in 15‐ml organ baths filled with oxygenated Krebs buffer solution at 37°C. After equilibration for at least 30 min and a wash every 15 min, the vessel segments were stretched to a stable tension of about 15 mN, with the optimal pre‐tension as determined earlier (Labruijere et al., 2015). Changes in tissue tension were measured with an isometric force transducer (Harvard, South Natick, MA, USA) and recorded on a flatbed recorder (Servogor 124, Goerz, Neudorf, Austria).

The distal coronary, internal mammary, and middle meningeal arteries were cut into circular 1‐ 2‐mm‐long segments and mounted in Mulvany myographs (Danish Myo Technology, Aarhus, Denmark) between two parallel small stainless‐steel wires (40‐μm calibre). All the baths were filled with warm Krebs buffer (37°C) and aerated with carbogen. The tension was normalized to 90% of l_100_ for all segments, the diameter when transmural pressure equals 100 mm Hg (Mulvany & Halpern, 1977). Data of these vessels were recorded using a LabChart data acquisition system (AD Instruments Ltd, Oxford, UK).

#### 2.4.1 Experimental protocols

A paired parallel set up (*i.e.,* all compounds were tested in different segments obtained from the same artery) was used. Initially, all segments were exposed to 30‐mM KCl to “prime” the tissue for stable contractions. After washout, the tissue was exposed to 100‐mM KCl to determine the maximal contractile response. After further washout, a concentration–response curve to vehicle, sumatriptan, or lasmiditan was constructed, using whole logarithmic steps from 1 nM up to 10 μM. After finishing the curve and washing several times until reaching equilibrium, the functional integrity of the endothelium was verified by observing relaxation to https://www.guidetopharmacology.org/GRAC/LigandDisplayForward?ligandId=2098 (10 nM; coronary and meningeal arteries) or https://www.guidetopharmacology.org/GRAC/LigandDisplayForward?ligandId=649 (1 μM; mammary arteries), after precontraction with the TxA_2_ analogue https://www.guidetopharmacology.org/GRAC/LigandDisplayForward?ligandId=1888 (10–100 nM; Chan et al., 2014; Labruijere et al., 2015).

Furthermore, in the internal mammary arteries, a concentration–response curve to lasmiditan and sumatriptan was also constructed after adding threshold concentrations of U46619 (*i.e.,* concentrations eliciting a contraction of ~10% of 100‐mM KCl response, determined in quarter logarithmic steps), used to unmask contractile properties of some agonists in the presence of an increased tension, as previously described (MaassenVanDenBrink et al., 2000). These contractile responses were evaluated post hoc in the absence (relaxation to bradykinin <18%) or presence (relaxation to bradykinin >18%) of functional endothelium; for this, endothelial function data was divided in percentiles, where values below the 50th percentile were considered without endothelium, and above the 50th percentile were considered with endothelium. Also, segments were preincubated with clinically relevant concentrations of sumatriptan (0.3 μM) or lasmiditan (1 μM) and followed by a concentration–response curve to lasmiditan or sumatriptan, respectively, to evaluate the possible interactions (*i.e.,* augmented vasoconstriction) between agonists. The clinically relevant concentration of sumatriptan was calculated as previously described (Labruijere et al., 2015); in the case of lasmiditan, it was estimated based on the *C*_max_ observed in humans following a 100‐mg dose (0.25 μM; Kovalchin, Ghiglieri, Zanelli, Ings, & Mathers, 2016).

### 2.5 Correlation between binding (pK_i_) and the contractile potency of lasmiditan and other triptans

We related previous (MaassenVanDenBrink et al., 1998; MaassenVanDenBrink, Reekers, Bax, & Saxena, 2000; Parsons et al., 1998; van den Broek et al., 2002) and current data obtained (see Section 3) to the potency of these compounds to contract the human isolated coronary artery. In case a compound failed to contract the human coronary artery, a fixed pEC_50_ value of 5 was set (see Table S3). Our pK_i_ values used for this correlation are in agreement with those previously published in the literature (Table S4; Figure S1).

### 2.6 Animal preparations

Although *in vitro* experiments with human isolated arteries provide invaluable information on vasoconstrictive responses in specific vascular beds, to discard haemodynamic changes after systemic administration of novel experimental therapeutic compounds, an *in vivo* model is necessary. The beagle dog is a well‐accepted species that has been in use for several years to predict human cardiovascular responses to novel experimental therapeutic compounds (Cason, Verrier, London, Mangano, & Hickey, 1987). Therefore, a total of 18 adult female beagle dogs (*Canis familiaris*) were selected from the CorDynamics, Inc. animal colony. These animals were obtained from Marshall BioResources (North Rose, NY, USA). Upon receipt at the Biologic Resources Laboratory (BRL) of the University of Illinois‐Chicago, dogs were examined by BRL veterinary personnel to ensure acceptable health status. Veterinary care was provided by the veterinarians and staff employed by the BRL. Dogs were acclimatized for at least 7 days prior to use and were pair‐housed in runs (meeting the size requirement set forth by the USDA Animal Welfare Act) with various cage‐enrichment devices. Room temperature set at 18–27°C, humidity at 30–70%, and fluorescent lights timed to give a 12 hr‐light and 12 hr‐dark cycle. Harlan Certified Canine food (25% Protein Diet #2025C, Harlan Teklad, Madison, WI, USA) was fed daily (500 g·day^−1^), and water was freely available in their runs. At the day of their terminal experiment, the animals were 10.0 to 11.5 months old, and their body weights ranged from 5.4 to 7.9 kg. Body weights were measured twice (approximately 1 week between measurements) prior to each animal's terminal procedure. Dogs were fasted for 16–18 hr prior to dosing.

All experimental protocols were approved and conducted by CorDynamics in compliance with the U.S. FDA Good Laboratory Practice guidelines (21 CFR Part 58), the Animal Welfare Act, the Guide for the Care and Use of Laboratory Animals, and the Office of Laboratory Animal Welfare. Animal studies are reported in compliance with the ARRIVE guidelines (Kilkenny, Browne, Cuthill, Emerson, & Altman, 2010) and with the recommendations made by the *British Journal of Pharmacology* (Curtis et al., 2018) and the editorial on reporting animal studies (McGrath & Lilley, 2015). For more specific details on design and statistical analysis, see Sections 2.7 and 2.8.

#### 2.6.1 General methods

Dogs were dosed with morphine s.c. (1 mg·kg^−1^) approximately 10–20 min prior to administration of propofol anaesthesia i.v. (5–6 mg·kg^−1^) to allow tracheal intubation. They were placed on a ventilator with isoflurane delivered at 1–2% in oxygen to maintain anaesthesia throughout the experiment, and s.c. morphine (0.5 mg·kg^−1^) was administered approximately every 2 hr while under anaesthesia. The local anaesthetic bupivacaine was infiltrated into the incision sites. A continuous 0.9% NaCl solution for injection drip (approximately 10 ml·kg^−1^·hr^−1^) was maintained until the start of dosing at which time it was discontinued. Dogs were placed on a heating pad set to maintain the animal's body temperature at approximately 37°C, and their body temperature was monitored throughout the experiment by placing a rectal temperature probe. Additionally, surface ECG leads were placed for anaesthesia monitoring throughout the experimental protocol.

A mid‐lateral neck incision was made and the left common carotid artery was exposed. A Transonic Systems Inc. (Ithaca, NY, USA) blood flow probe and two Sonometric Corporation (London, Ontario, Canada) crystals for arterial dimensional analysis were affixed to the artery. A left lateral thoracotomy (sixth, intercostal space) was performed, and the left circumflex (LCX) coronary artery was exposed. A Transonic Systems Inc. blood flow probe and two Sonometric Corporation crystals for arterial dimensional analysis were affixed to the artery. A solid‐state high‐fidelity pressure catheter (Millar Inc., Houston, TX, USA) for measurement of arterial pressure (mean, MAP; systolic, SAP; and diastolic, DAP) and heart rate was inserted into a femoral artery and secured in place with silk suture. An indwelling catheter was placed into the femoral vein for collecting blood (2 ml) prior to the start of dosing and at the end of each 20 min infusion period (see experimental protocol) for bioanalytical analysis.

#### 2.6.2 Experimental protocol

Upon completion of the general instrumentation, a 15‐min equilibrium period was allowed for a stable haemodynamic condition. Baseline values (defined as the average of the three 5‐min values at the aforementioned 15 min) of MAP, heart rate, and carotid and LCX coronary artery diameter and flow were determined. Subsequently, the 18 dogs were randomly assigned into three groups (*n* = 6 each), which received vehicle (saline), lasmiditan (0.03, 0.13, 1.13, 4.13, and 11.13 mg·kg^−1^), or sumatriptan (0.03–11.3 mg·kg^−1^) respectively. All treatments were filtered through a 0.2‐μM membrane and administered i.v. in the escalating cumulative doses mentioned above. Dose intervals amongst different treatments were administered each 20 min. At the end of the experiment, dogs were killed while under anaesthesia via a barbiturate overdose.

### 2.7 Sample size calculation, randomization, and blinding

#### 2.7.1 Sample size calculation

The animal sample size (*n* = 6 each group) was calculated by CorDynamics based on their previous studies (Cushing et al., 2009). For the *in vitro* studies, we based the experimental number (*n* = 5–7 each group) on previous studies from our group (Chan, Baun, et al., 2011; Labruijere et al., 2015).

#### 2.7.2 Randomization

For the *in vitro* experiments, all artery segments were cut into rings and randomly assigned to a bath, and then the treatment group was designed by using a table of random numbers.

For the *in vivo* experiments, the animals initially divided in sets (*n* = 6 each group as described above) were randomly assigned to study groups by CorDynamics staff.

#### 2.7.3 Blinding

For the radioligand and second messenger assays, the analyst was not blinded to the compounds but to the research hypothesis. For the vascular *in vitro* experiments, values were calculated using the dose–response auto‐analyse selection feature of LabChart. During the analysis, the investigator was unaware of which concentration response curve was being analysed. The *in vivo* experimental values (*i.e.,* the changes in MAP or artery diameter) in each group of animals were simultaneously obtained by at least two different CorDynamics investigators, with at least one of the investigators blinded.

### 2.8 Data presentation and statistical analysis

All data in the text and figures are presented as the mean ± SEM from (*n*) experiments, as shown in the figure legends. The data and statistical analysis comply with the recommendations of the *British Journal of Pharmacology* on experimental design and analysis in pharmacology (Curtis et al., 2018).

#### 2.8.1 Radioligand binding and second messenger activity

For binding competition and second messenger activity assays, reference compounds were tested at several concentrations in duplicate to obtain a concentration–response curve, and an estimated pEC_50_ (negative logarithm of the concentration eliciting 50% of the maximal contractile response, *i.e.,* *E*_max_) or pIC_50_ value (negative logarithm of the concentration that displaced 50% of the radioligand) was calculated using XLFit (IDBS, Guildford, UK). Additionally, the reference values obtained were compared to historical values obtained from the same receptor and used to validate the experimental session. A session was considered as valid only if the reference value was found to be within a 0.5 log interval from the historical value, for assays where historical values (determined in at least five experiments) were available (Abourashed, Koetter, & Brattström, 2004; Barac et al., 2012; Sun, Blanton, Gabriel, & Canney, 2005). For the new assays developed in this study (*i.e.,* 5‐ht_1E_ receptor), the two independent pIC_50_ determined must be concordant with a 1 log unit interval for the assays to be validated. When less than 50% inhibition of binding or second messenger activation was obtained at 10 μM, a pIC_50_/pEC_50_ of “<5” was set

#### 2.8.2 Experiments with human isolated arteries

For the *in vitro* studies using human blood vessels, concentration–response curves were analysed using GraphPad software (GraphPad Software Inc., San Diego, CA, USA; RRID:SCR_002798) to determine pEC_50_ values as previously reported (Labruijere et al., 2015). When a plateau in the concentration–response curve was not reached, the response observed with the highest concentration used (*i.e.,* 100 μM) was considered as *E*_max_. Differences between pEC_50_ and *E*_max_ values of the compounds were evaluated with Tukey's test, once an ANOVA for paired data had revealed that the samples represented different populations. Values of *P* < .05 were considered to indicate significant differences.

#### 2.8.3 *In vivo* studies

In the *in vivo* studies, each haemodynamic parameter was analysed with a repeated measure analysis of covariance (RANCOVA) for changes from baseline at time intervals of 5, 10, 15, and 20 min for each of the five dose levels. The model factored the treatment (TRT), the time after dose (TIME), and the interaction of time after dose with treatment (TRT * TIME). The SAS® procedure PROC MIXED was used for analysis with TIME as the repeated effect and ANIMAL as the subject. The covariance between errors from the same animal at different time points was selected based on the corrected Akaike's information criterion from selected covariance structures of VC, AR(1), UN, and CS. Non‐monotonic dose–responses were evaluated. Within the framework of the RANCOVA, comparisons were made for vehicle‐ versus lasmiditan‐treated animals and for vehicle‐ versus sumatriptan‐treated animals. If TRT * TIME was significant, the comparisons were conducted for each time interval using an analysis of covariance (ANCOVA) model with an effect for treatment and baseline as a covariate. If only the TRT effect was significant, the comparison was conducted across the pooled time intervals for the overall phase only. These non‐monotonic treatment group comparisons were conducted at the *P* = .01 significance level. Baseline data were analysed with an ANOVA for each time interval. Factors in the model included treatment (TRT). All statistical analyses were conducted with SAS® version 9.2 (RRID:SCR_008567). After the database lock, post hoc analyses for coronary artery diameter and carotid artery diameter (primary endpoints) at the clinically relevant time interval 20 min (completion of dose administration) for each cumulated dose (0.03–11.13 mg·kg^−1^) were performed for comparisons between sumatriptan and vehicle. A significance level of *P* ≤ .025 was used for the RANCOVA using Bonferroni correction of two tests (coronary and carotid artery diameter).

### 2.9 Materials

The compounds used in the present study (obtained from the sources indicated) were 5‐HT hydrochloride, naratriptan hydrochloride, almotriptan malate, avitriptan fumarate, and sumatriptan succinate (Sigma Chemical Co., St. Louis, MO, USA); lasmiditan hemisuccinate (Eli Lilly & Co., Indianapolis, IN, USA); 5‐CT maleate, sumatriptan succinate, zolmitriptan, rizatriptan benzoate, eletriptan hydrobromide, donitriptan hydrochloride, LY334370 hydrochloride, and LY344864 hydrochloride (Tocris Bioscience Co., Park Ellisville, MO, USA); ergotamine tartrate (TEVA Pharmaceutical Industries Ltd., Petach Tivka, Israel); and alniditan salt (kind gift of Janssen Pharmaceutica, Beerse, Belgium).

All compounds were dissolved in distilled water or physiological saline for the *in vitro* and *in vivo* studies respectively. These vehicles had no effect on the baseline MAP values or artery diameter (not shown). Fresh solutions were prepared for each experiment. The doses mentioned in this text refer to the free base of substances.

### 2.10 Nomenclature of targets and ligands

Key protein targets and ligands in this article are hyperlinked to corresponding entries in http://www.guidetopharmacology.org, the common portal for data from the IUPHAR/BPS Guide to PHARMACOLOGY (Harding et al., 2018), and are permanently archived in the Concise Guide to PHARMACOLOGY 2017/18 (Alexander et al., 2017).

## 3 RESULTS

### 3.1 Pharmacological characterization of lasmiditan

**Figure 1:**
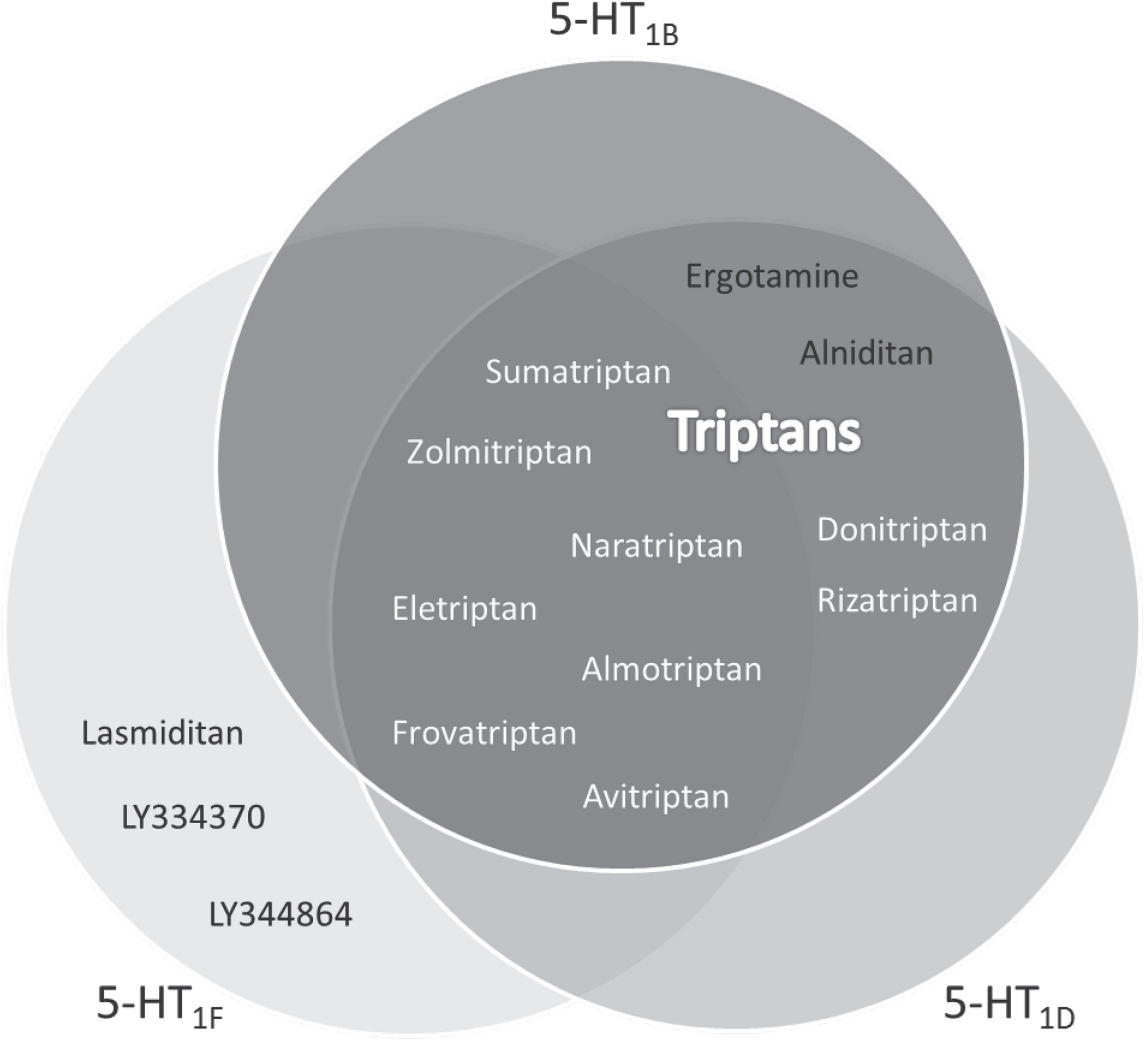
Summary of the agonist profiles (pEC_50_ > 7) of the antimigraine drugs tested on the 5‐HT_1B_, 5‐HT_1D_, and 5‐HT_1F_ receptors. Redrawn from Rubio‐Beltran, Labastida‐Ramirez, Villalon, and MaassenVanDenBrink (2018)

As shown in Table 1, radioligand studies revealed that lasmiditan selectively binds to the human 5‐HT_1F_ receptor. On the other hand, the triptans almotriptan, avitriptan, eletriptan, frovatriptan, naratriptan, sumatriptan, and zolmitriptan showed affinity also for 5‐HT_1B_, 5‐HT_1D_, and 5‐HT_1F_ receptors, while alniditan, donitriptan, ergotamine, and rizatriptan had affinity for 5‐HT_1B_ and 5‐HT_1D_ receptors. Most importantly, when analysing their second messenger activity, we observed that ergotamine is an agonist of the 5‐HT_1A/B/D_, 5‐HT_2A/B_, and 5‐HT_7_ receptors (but not of 5‐HT_1F_ receptors). Similarly, sumatriptan, zolmitriptan, naratriptan, almotriptan, eletriptan, frovatriptan, and avitriptan are agonists of the 5‐HT_1B/D_ receptors and also of the 5‐HT_1F_ receptor. Lasmiditan, as well as LY344864, displayed a high potency only for the 5‐HT_1F_ receptor (Table 2). In Figure 1, the agonistic profile of the different antimigraine drugs tested on the 5‐HT_1B/D/F_ receptors (*i.e.,* relevant for migraine therapy) are represented. When comparing our results with those previously published, our values are in agreement with those in the literature (see Tables S4–S5; Figure S1). Moreover, no functional responses to 5‐CT were observed in the CHO‐K1 cells transfected with an unrelated GPCR (Figure S2).

**Table 1:** Summary of pIC_50_ (negative logarithm of the molar concentration of these compounds at which 50% of the radioligand is displaced) and pK_i_ (negative logarithm of the molar concentration of the *K*_i_) values of individual antimigraine drugs at 5‐HT receptors

Agonist	5‐HT_1A_		5‐HT_1B_		5‐HT_1D_		5‐ht_1E_		5‐HT_1F_		5‐HT_2A_		5‐HT_2B_		5‐HT_7_	
	pIC_50_	pK_i_	pIC_50_	pK_i_	pIC_50_	pK_i_	pIC_50_	pK_i_	pIC_50_	pK_i_	pIC_50_	pK_i_	pIC_50_	pK_i_	pIC_50_	pK_i_
Ergotamine tartrate	9.19	9.70	8.87	9.34	8.63	9.31	6.08	6.39	6.71	7.13	7.62	8.14	7.73	7.94	7.13	7.23
Sumatriptan succinate	6.63	7.14	7.81	8.29	8.31	9.00	5.42	5.72	7.13	7.55	<5	<5	<5	<5	6.10	6.19
Zolmitriptan	7.28	7.79	8.85	9.33	9.28	9.97	7.51	7.81	7.13	7.55	<5	<5	<5	<5	6.97	7.06
Naratriptan hydrochloride	7.31	7.82	8.75	9.22	8.62	9.30	7.83	8.13	8.33	8.75	<5	<5	<5	5.08	5.84	5.93
Rizatriptan benzoate	6.81	7.32	7.51	7.99	8.15	8.83	6.48	6.78	6.4	6.82	<5	<5	5.30	5.51	<5	<5
Almotriptan malate	6.23	6.73	7.97	8.45	7.57	8.26	<5	<5	7.15	7.57	<5	<5	<5	<5	6.36	6.46
Eletriptan hydrobromide	8.20	8.71	8.80	9.28	9.31	9.99	6.91	7.21	7.35	7.77	5.42	5.94	6.14	6.35	6.61	6.70
Frovatriptan racemate	6.83	7.34	8.09	8.57	8.10	8.78	<5	5.18	6.50	6.92	<5	<5	<5	<5	6.88	6.97
Donitriptan hydrochloride	7.42	7.93	9.29	9.77	9.18	9.86	5.47	5.77	<5	5.18	5.83	6.35	5.88	6.09	6.12	6.21
Avitriptan fumarate	7.20	7.71	8.32	8.80	8.42	9.11	5.15	5.45	6.69	7.11	5.11	5.63	5.73	5.94	6.03	6.12
Alniditan dihydrochloride	8.81	9.32	8.93	9.41	8.66	9.35	5.98	6.28	6.02	6.44	<5	5.43	6.67	6.88	7.16	7.26
Lasmiditan hemisuccinate	5.88	6.39	5.54	6.02	5.62	6.31	5.54	5.84	8.09	8.51	<5	<5	5.01	5.22	<5	<5
LY334370 hydrochloride	7.98	8.49	6.74	7.21	6.24	6.92	6.83	7.13	9.03	9.45	5.11	5.63	5.98	6.19	5.66	5.75
LY344864 hydrochloride	6.12	6.63	6.13	6.61	5.83	6.52	6.05	6.35	8.38	8.80	5.11	5.63	5.31	5.52	5.69	5.78

*Note*. The lesser than symbol (<) indicates that less than 50% inhibition of binding was obtained at 10 μM. The radioligands used and their concentrations are described in Table S1.

**Table 2:** Summary of pEC_50_ values of cAMP (5‐HT_1A/B/E/F_ and 5‐HT_7_), GTPγS (5‐HT_1A/B/D/E/F_), and IP (5‐HT_2_) assays of individual antimigraine drugs at 5‐HT receptors

Agonist	5‐HT_1A_		5‐HT_1B_		5‐HT_1D_	5‐ht_1E_		5‐HT_1F_		5‐HT_2A_	5‐HT_2B_	5‐HT_7_
	cAMP	GTPγS	cAMP	GTPγS	GTPγS	cAMP	GTPγS	cAMP	GTPγS	IP	IP	cAMP
Ergotamine tartrate	9.78	9.63	9.94	9.52	9.43	5.95	5.74	5.97	6.30	9.25	8.72	7.09
Sumatriptan succinate	<5	<5	7.32	7.91	8.30	5.99	5.79	8.03	6.80	<5	<5	5.22
Zolmitriptan	<5	5.52	7.87	8.42	9.51	8.18	7.81	8.00	6.67	<5	<5	6.28
Naratriptan hydrochloride	<5	6.52	8.05	8.86	8.80	7.75	8.17	8.38	8.05	<5	<5	<5
Rizatriptan benzoate	<5	<5	7.08	7.56	8.11	7.34	6.90	6.54	5.91	<5	5.49	<5
Almotriptan malate	<5	5.48	7.08	7.85	7.75	<5	<5	7.79	6.90	<5	5.20	<5
Eletriptan hydrobromide	5.74	6.38	8.00	8.09	9.04	7.53	6.90	8.13	6.88	6.07	6.81	6.45
Frovatriptan racemate	<5	6.12	7.98	8.14	8.36	5.04	<5	7.10	6.35	<5	<5	7.42
Donitriptan hydrochloride	5.94	6.74	9.96	9.52	9.51	<5	<5	<5	<5	8.10	7.61	5.23
Avitriptan fumarate	<5	6.19	8.57	8.68	9.27	5.52	<5	7.09	6.05	6.91	6.41	5.38
Alniditan dihydrochloride	7.00	7.29	8.87	8.90	8.20	5.68	5.21	5.92	5.17	<5	7.15	6.32
Lasmiditan hemisuccinate	<5	<5	<5	<5	6.64	6.17	5.34	8.43	7.80	<5	<5	<5
LY334370 hydrochloride	5.84	6.96	6.52	5.80	6.92	7.53	6.95	9.08	9.38	<5	<5	<5
LY344864 hydrochloride	<5	<5	<5	5.82	6.93	6.22	6.12	8.72	7.85	<5	<5	<5

*Note*. These values represent the negative logarithm of the molar concentration of these compounds at which 50% of their maximal response is exerted. The lesser than symbol (<) indicates that less than 50% response was obtained at 10 μM. The reference compounds used and their concentrations are described in Table S2.

### 3.2 Human isolated arteries

**Figure 2:**
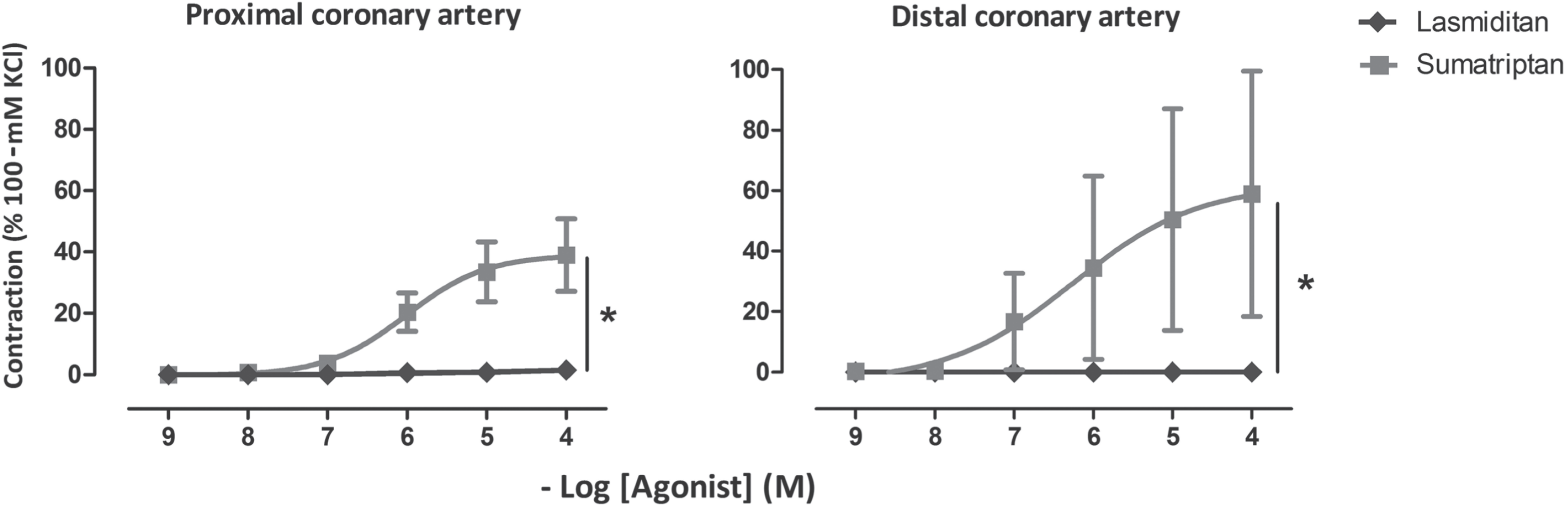
Contractile responses to lasmiditan and sumatriptan (1 nM–100 μM) in the isolated human proximal (left) and distal (right) coronary arteries; *P < .05, significantly different as indicated; *n* = 6 each

In the human isolated coronary arteries, sumatriptan induced significant contractions in a concentration‐dependent manner in the proximal (*E*_max_ 39 ± 12%, pEC_50_ 6.4 ± 0.2; *n* = 6) and distal (*E*_max_ 59 ± 41%, pEC_50_ 6.02 ± 0.2; n = 6) coronary segments, even at clinically relevant concentrations (see Figure 2). In contrast, as compared with vehicle, lasmiditan was devoid of any significant contractile effects in both coronary segments, even at its highest concentration (100 μM, supratherapeutic; *E*_max_ 1 ± 1%).

**Figure 3:**
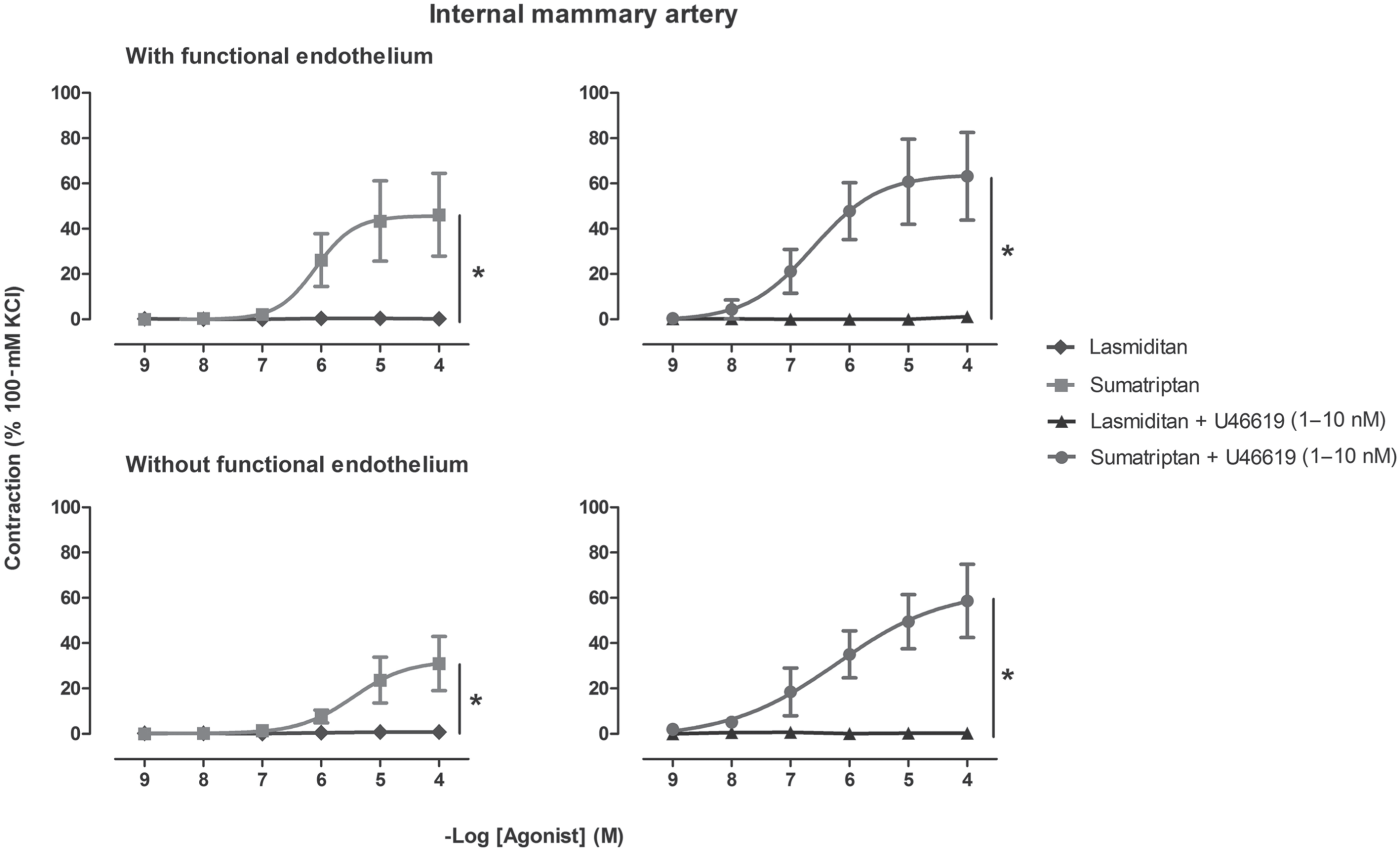
Contractile responses to sumatriptan and lasmiditan (1 nM–100 μM) in the absence (left) and presence (right) of a threshold precontraction with U46619 (1–10 nM) in the isolated human internal mammary arteries with (upper panel, *n* = 5) and without (lower panel, *n* = 7) functional endothelium; *P < .05, significantly different as indicated

Moreover, Figure 3 shows the effects of lasmiditan and sumatriptan on internal mammary arteries in the absence and presence of endothelium. Sumatriptan induced concentration‐dependent contractions in blood vessels with (*E*_max_ 46 ± 18%, pEC_50_ 6.07 ± 0.07; *n* = 5) and without functional endothelium (*E*_max_ 31 ± 12%, pEC_50_ 5.6 ± 0.94; *n* = 7). After precontraction with threshold concentrations of U46619, sumatriptan also produced concentration‐dependent contractions in the presence (*E*_max_ 63 ± 19%, pEC_50_ 6.83 ± 0.05; *n* = 5) and absence (*E*_max_ 59 ± 16%, pEC_50_ 6.02 ± 0.59; *n* = 7) of functional endothelium. In marked contrast, vehicle and lasmiditan were devoid of any significant contractile effects in internal mammary arteries (*E*_max_ 1 ± 1% without endothelium; *E*_max_ 0 ± 0% with endothelium), even after a modest precontraction with U46619 (*E*_max_ 0 ± 1% without endothelium; *E*_max_ 1 ± 1% with endothelium).

**Figure 4:**
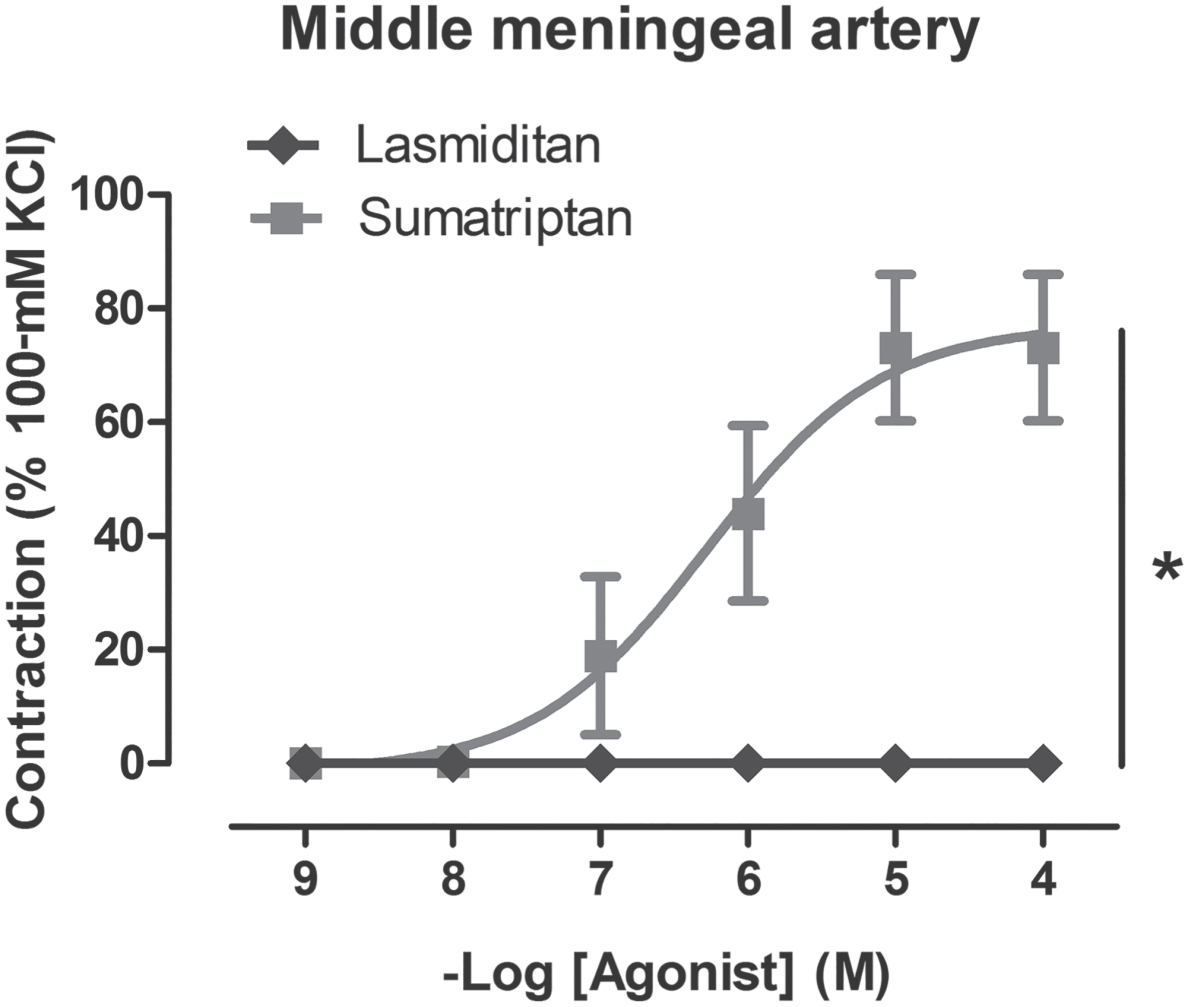
Contractile responses to sumatriptan and lasmiditan (1 nM–100 μM) in the isolated human middle meningeal arteries; *P < .05, significantly different as indicated; *n* = 6 each

In the middle meningeal artery, sumatriptan induced significant concentration‐dependent contractions (*E*_max_ 73 ± 13%, pEC_50_ 6.32 ± 0.15; *n* = 6), whereas lasmiditan did not induce any significant contraction at all concentrations tested (*E*_max_ 0 ± 0%, Figure 4).

## 4 INTERACTION EXPERIMENTS

**Figure 5:**
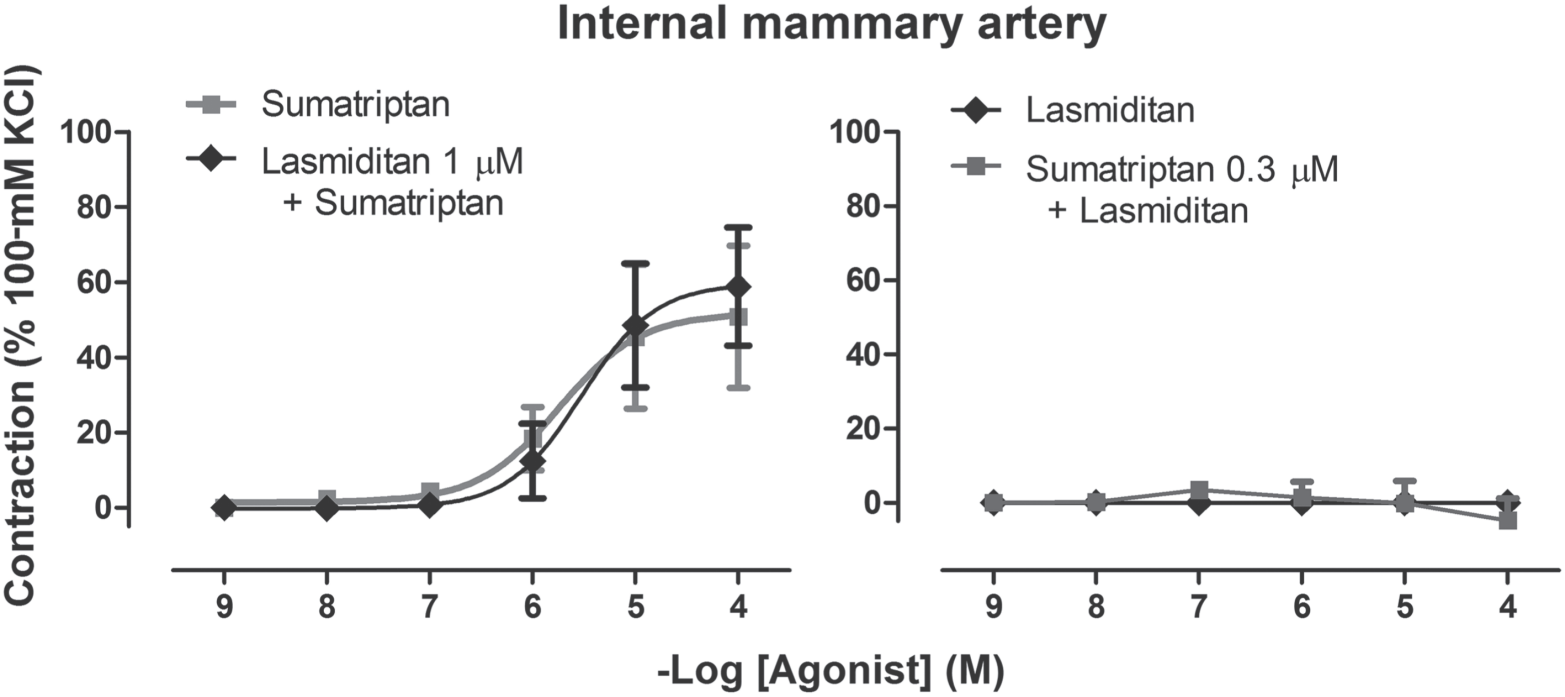
Contractile responses to sumatriptan and lasmiditan (1 nM–100 μM) in the internal mammary artery, after preincubation with the clinically relevant concentration of sumatriptan (0.3 μM) or lasmiditan (1 μM), and followed by a concentration–response curve to lasmiditan or sumatriptan, respectively (*n* = 6 each)

As shown in Figure 5, in the internal mammary arteries, after preincubation with lasmiditan (1 μM), no changes in the contractile responses to sumatriptan were observed when compared to the concentration–response curve to sumatriptan alone (*E*_max_ 59 ± 16%, pEC_50_ 5.34 ± 0.1 vs. *E*_max_ 51 ± 19%, pEC_50_ 5.71 ± 0.7; *n* = 5 each). In addition, the highest concentration of lasmiditan produced a non‐significant vasodilation when preincubated with sumatriptan's clinically relevant concentration (0.3 μM) when compared to the concentration–response curve to lasmiditan without sumatriptan (*E*_max_ − 4.8 ± 5.95% vs. *E*_max_ 0 ± 0%; *n* = 5 each).

### 4.1 Correlation between binding (pK_i_) and the contractile potency of lasmiditan and other triptans

**Figure 6:**
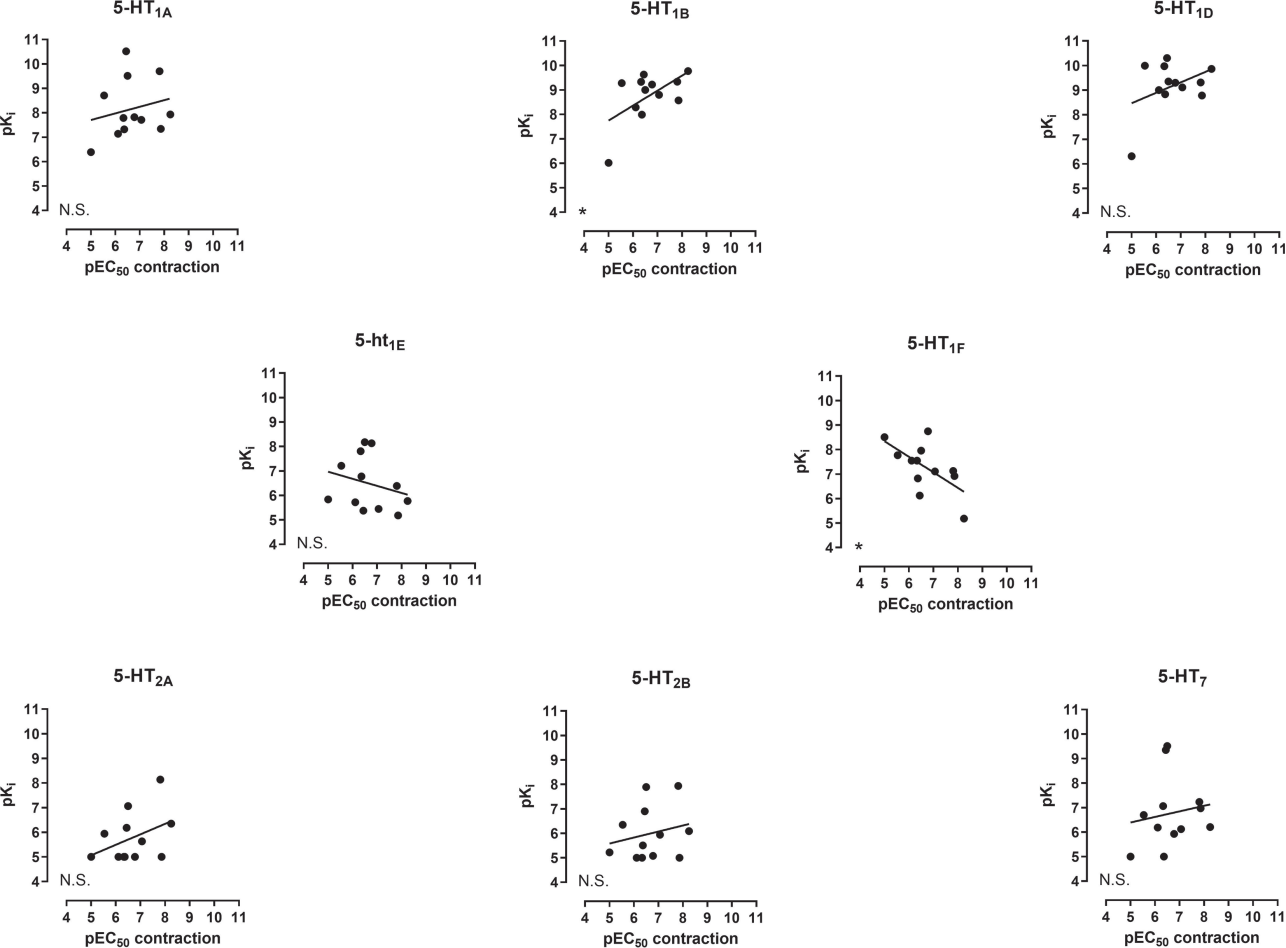
Correlation between the pK_i_ values obtained in our study and the contractile potency of lasmiditan, triptans (sumatriptan, zolmitriptan, naratriptan, rizatriptan, eletriptan, frovatriptan, donitriptan, and avitriptan), and other 5‐HT receptors ligands (ergotamine, alniditan, 5­HT, and 5‐carboxamidotryptamine) in human isolated coronary arteries; N.S., non‐significant; ^*^ *P* < .05, significant correlation

As shown in Figure 6, the potency of the compounds tested to contract the human isolated coronary artery was positively correlated with their potency to bind the 5‐HT_1B_ receptor, whereas it was negatively correlated for the 5‐HT_1F_ receptor. This was also observed when correlating the pEC_50_ values obtained in our second messenger assays and the contractile potency of the compounds tested in the human coronary arteries (Figure S3).

### 4.2 *In vivo* studies

In anaesthetised dogs, a directly proportional relationship was observed between lasmiditan and sumatriptan cumulative i.v. doses and their plasma concentrations; these latter values were used for validating the concentrations used in the *in vitro* studies (data not shown).

**Figure 7:**
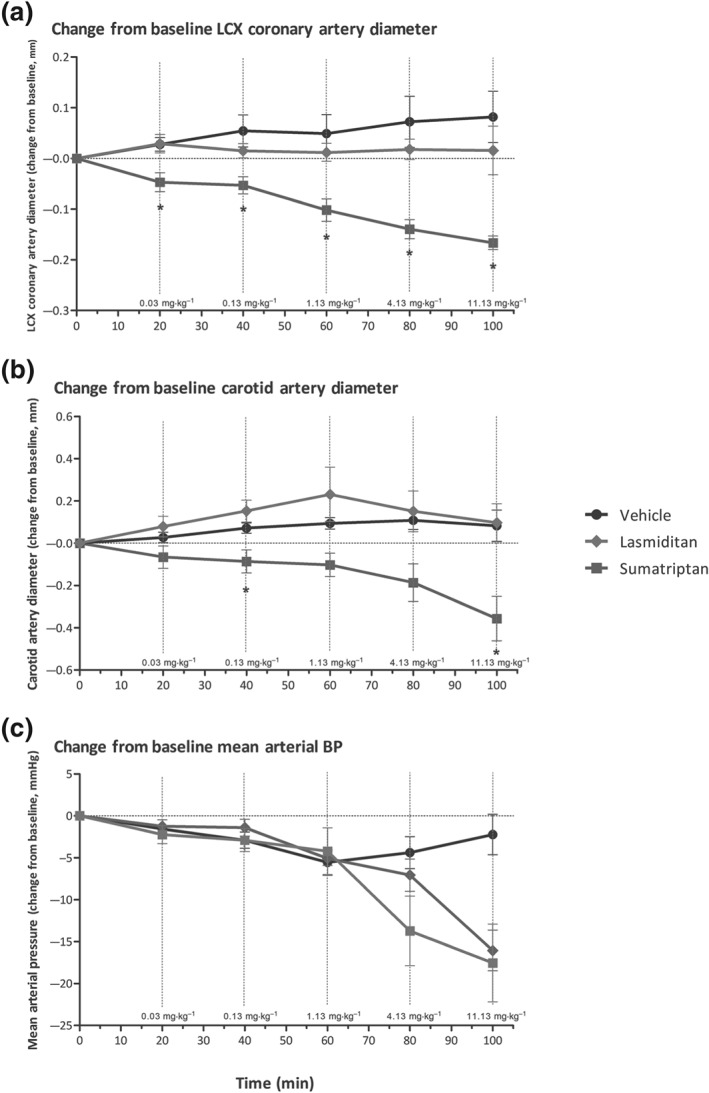
Changes in the left circumflex (LCX) coronary artery diameter (a), carotid artery diameter (b), and mean arterial blood pressure (c) after the continuous infusion of lasmiditan and sumatriptan (0.03–11.13 mg·kg^−1^ each) or the corresponding infusion volumes of vehicle in female beagle dogs (*n* = 6 each). *P < .05, significantly different from vehicle; post hoc analysis (see methods)

Moreover, as shown in Figure 7, changes in carotid artery diameter were not statistically significant in the lasmiditan‐treated group as compared to the time‐matched vehicle control group. In contrast, as expected, sumatriptan induced dose‐dependent decreases in carotid artery diameter, although these effects were statistically significant only at the doses of 0.13 mg·kg^−1^ (clinically relevant) and 11.13 mg·kg^−1^ (Figure 7). In the LCX coronary artery, lasmiditan failed to induce any statistically significant change in diameter at any dose. Conversely, statistically significant decreases in the LCX coronary artery diameter were observed at all doses in sumatriptan‐treated animals as compared to the time‐matched vehicle control animals, even at the lowest dose of 0.03 mg·kg^−1^, which already corresponds to a clinically relevant dose.

Carotid blood flow was not significantly different after vehicle or clinically relevant doses of lasmiditan (0.03–1.13 mg·kg^−1^). Lasmiditan decreased carotid blood flow significantly, but only after the supratherapeutic cumulative doses of 4.13 and 11.13 mg·kg^−1^. In contrast, sumatriptan elicited a statistically significant rapid, dose‐dependent, decrease in carotid blood flow at all doses tested. Regarding coronary blood flow, the administration of vehicle, lasmiditan, or sumatriptan did not elicit any statistically significant changes (data not shown).

Heart rate was stable over the course of the study, and no significant changes were observed in the lasmiditan or vehicle groups. In the sumatriptan‐treated group, cumulative doses of 4.13 and 11.13 mg·kg^−1^ elicited dose‐dependent decreases in heart rate, which were statistically significant, with a peak decrease at 90 min of 16.5 ± 6 bpm (data not shown). MAP, SAP, and DAP showed no significant changes in either sumatriptan or lasmiditan‐treated groups as compared to the time‐matched vehicle group at cumulative doses of up to 4.13 mg·kg^−1^. At higher doses, both lasmiditan‐ and sumatriptan‐treated groups showed a dose‐dependent trend to decrease MAP, SAP, and DAP; however, these changes were not statistically significant (Figure 7).

## 5 DISCUSSION

The current study was designed to investigate the selectivity and vasoconstrictor profile of lasmiditan, which belongs to a novel class of acute antimigraine drugs, the ditans. According to its binding and functional activity, it was confirmed that lasmiditan is a highly selective agonist of the 5‐HT_1F_ receptor. Moreover, as lasmiditan was developed based on the premise that coronary vasoconstriction is a side effect of the triptans attributed to 5‐HT_1B_ receptors, we studied the vasoconstrictor potential of 5‐HT_1F_ agonism in two different vascular models and compared our *in vitro* and *in vivo* results to those obtained with sumatriptan, since this is the “gold standard” triptan for acute antimigraine treatment. This allowed us to compare the results from the current study with results obtained earlier. In accordance with our previous work (Chan et al., 2014), sumatriptan induced a concentration‐dependent contraction in human isolated coronary arteries, which tended to be larger in distal than in proximal coronary artery segments. This contraction was apparent at clinically relevant concentrations and is most likely due to activation of 5‐HT_1B_ receptors in vascular smooth muscle (Chan et al., 2014). In contrast, lasmiditan did not induce a contraction at concentrations up to 100 μM (≥100× the clinically relevant concentrations) in either proximal or distal coronary arteries. Although moderate to intense expression of mRNA encoding the 5‐HT_1F_ receptor in human coronary arteries has been described (Nilsson et al., 1999), the presence of mRNA does not necessarily mean protein expression, which may well be the case. Thus, the physiological role of this receptor in blood vessels remains to be determined.

Subsequently, we performed more in‐depth experiments in the internal mammary artery, where we studied the influence of endothelial functional quality, and the effects of a precontraction induced by the TxA_2_ analogue U46619, as such a precontraction is known to “unmask” or augment contractions to other ligands, such as sumatriptan (MaassenVanDenBrink, van den Broek, de Vries, Upton, et al., 2000). As observed in the coronary artery, sumatriptan contracted the internal mammary artery, similarly in both segments with and without functionally active endothelium. In accordance with earlier observations (MaassenVanDenBrink, van den Broek, de Vries, Upton, et al., 2000), the contractions to sumatriptan were augmented in the presence of U46619. In contrast, lasmiditan did not induce any contraction in the absence or presence of U46619 in either vessel segments with or without endothelium. Interestingly, in the rabbit saphenous vein, precontraction with https://www.guidetopharmacology.org/GRAC/LigandDisplayForward?ligandId=1884 unmasked a contractile response to the 5‐HT_1F_ receptor agonists, LY334370 and LY344864, but only after high concentrations (>10 μM), and therefore likely due to activation of vascular 5‐HT_1B_ receptors (Cohen & Schenck, 2000). Hence, the absence of contractile responses with high concentrations of lasmiditan, even in precontracted vessels, is surprising, given the difference in affinity between sumatriptan and lasmiditan to the 5‐HT_1B_ receptor. However, binding affinity does not always correlate with second messenger activation and biological response (Colquhoun, 1998). Therefore, while our radioligand studies (Table 1) showed a ~100‐fold binding difference to the 5‐HT_1B_ receptor between sumatriptan (pIC_50_ = 7.81) and lasmiditan (pIC_50_ = 5.54), our cAMP assays (Table 2) showed that the functional potency (pEC_50_) of sumatriptan was 7.32 and lasmiditan was <5. As we could not determine the precise pEC_50_ value of lasmiditan, the potency difference between both compounds could be larger than 1,000‐fold and thus explain the complete absence of vasoconstrictive responses even at supra‐therapeutic concentrations such as 100 μM. This could represent a cardiovascular safety advantage over its triptan predecessors.

Additionally, as contraction of the meningeal artery is thought to contribute to the antimigraine effects of the triptans (Benemei et al., 2017; Chan, Vermeersch, de Hoon, Villalón, & MaassenVanDenBrink, 2011; Rubio‐Beltran et al., 2018), but is not a class effect of all antimigraine drugs (e.g., gepants), we investigated the contractions to sumatriptan and lasmiditan in human meningeal arteries. In accordance to the previously described craniovascular selectivity of the triptans (MaassenVanDenBrink et al., 2000; van den Broek et al., 2002), contractions to sumatriptan were larger in this dural artery than those in the proximal coronary artery. However, as we have also previously shown, contractions to sumatriptan in distal coronary artery (and internal mammary artery) were not significantly different from those in meningeal artery (Chan et al., 2014). In contrast, lasmiditan was devoid of vascular effects in this cranial vessel. Therefore, the efficacy of lasmiditan as acute migraine treatment may be due to inhibition of https://www.guidetopharmacology.org/GRAC/LigandDisplayForward?ligandId=695 release from perivascular fibres or direct central (antinociceptive) modulation (Rubio‐Beltran et al., 2018).

Our binding studies showed that, as mentioned previously, most of the triptans available in the market, namely, almotriptan, frovatriptan, naratriptan, sumatriptan, and zolmitriptan, are also agonists of the 5‐HT_1F_ receptor (Figure 1). Furthermore, the correlation between binding and the contractile potency of the compounds tested revealed that the potency of the agonists to contract the HCA positively correlated to their potency to bind the 5‐HT_1B_ receptor, whereas it negatively correlated for the 5‐HT_1F_ receptor (Figure 6), and this was also observed when correlating the contractile potency and second messenger activation (Figure S3). Moreover, when analysing the correlation between second messenger activation by 5‐HT_1B_ versus 5‐HT_1F_ receptors, also a negative correlation was observed (Figure S4). These results, together with our *in vitro* data, suggest that although the mRNA of both receptor subtypes has been described in human vasculature (Bouchelet et al., 1996; Bouchelet et al., 2000; Chan et al., 2009; Chan et al., 2014; Nilsson et al., 1999; Parsons et al., 1998; van den Broek et al., 2002), only activation of the 5‐HT_1B_ receptor will result in vasoconstriction of, at least, coronary, mammary, and meningeal arteries, whereas activation of the 5‐ HT_1F_ receptor will not. This could suggest that either 5‐HT_1F_ receptors in human vasculature are not functional or that 5‐HT_1F_ receptor mRNA is not translated to protein.

When considering the acute haemodynamic effect of sumatriptan in humans, it is well known that after subcutaneous administration, there are vasopressor responses in the systemic arterial circulation and coronary artery vasoconstriction (MacIntyre, Bhargava, Hogg, Gemmill, & Hillis, 1993). Although we observed carotid and coronary vasoconstriction in anaesthetized dogs, there were no significant increases in blood pressure, as previously reported in this animal model (Villalón & Terrón, 1994). In fact, after high doses of sumatriptan, a non‐significant tendency to decrease blood pressure and significant decreases in heart rate were observed, most probably due to inhibition of vascular and cardiac sympathetic outflows via the stimulation of prejunctional 5‐HT_1B/1D_ receptors on perivascular (Villalón et al., 1998) and cardiac (Sánchez‐López et al., 2003; Sánchez‐López et al., 2004) sympathetic nerves. Lasmiditan only showed a trend to decrease blood pressure at the highest (supratherapeutic dose), which, based on the affinity of lasmiditan (see Table 1), could be due to a non‐selective activation of prejunctional 5‐HT_1D_ receptors and subsequent inhibition of sympathetic perivascular nerves (Villalón et al., 1998). Admittedly, this has not been shown directly in dogs but in pithed Wistar rats, and, in patients, no changes in blood pressure have been observed (Farkkila et al., 2012; Ferrari et al., 2010). Further experiments, falling beyond the scope of the present study, would be required to shed more light on the mechanisms behind these responses, which are only observed at non‐clinically relevant doses.

In summary, our *in vitro* and *in vivo* results indicate that lasmiditan is devoid of contractile properties in isolated human and anaesthetized dog arteries respectively. This might be of particular relevance in migraine patients who have a high risk of developing cardiovascular disease, such as subjects with hemiplegic migraine, prolonged migraine with aura, or with established cardiovascular disease. Clearly, further studies are needed to evaluate the safety of lasmiditan in these specific patient populations and its effectiveness compared with triptans. Finally, clinical trials have shown that lasmiditan is effective for migraine treatment (Goadsby et al., 2019; Kuca et al., 2018), suggesting a mechanism of action (partly) different to that of the triptans (Rubio‐Beltran et al., 2018).

In conclusion, our data support our initial hypothesis that lasmiditan is a high‐affinity and highly selective agonist for the *human* 5‐HT_1F_ receptor that is devoid of contractile properties in human isolated blood vessels and in anaesthetized canines.

## AUTHOR CONTRIBUTIONS

E.R.B., E.Z., L.M., A.H.J.D., M.R.G., P.B.S., K.W.J., J.K., C.M.V., and A.M.v.d.B. contributed to conception and design. E.R.B., A.L.R., K.A.H., L.M., M.R.G., and P.B.S. performed the acquisition, analysis, and interpretation of data. E.R.B. and A.M.v.d.B. drafted the manuscript. E.R.B., A.L.R., K.A.H., A.B., A.J.J.C.B., E.Z., L.M., A.H.J.D., M.R.G., P.B.S., K.W.J., J.K., C.M.V., and A.M.v.d.B. revised the manuscript. All authors approved the final version of the manuscript.

## CONFLICT OF INTEREST

E.R.B. and A.L.R. received travel support from Eli Lilly/CoLucid. E.Z. and C.M.V. received consultation fees from Eli Lilly/CoLucid. L.M., M.R.G. and P.B.S. performed experiments under a research contract with Eli Lilly/CoLucid. J.K. is former employee of Eli Lilly/CoLucid. K.W.J. is employee of Eli Lilly. A.M.v.d.B. received research grants and/or consultation fees from Amgen/Novartis, Eli Lilly/CoLucid, Teva, and ATI. All other authors declare no conflicts of interest.

## DECLARATION OF TRANSPARENCY AND SCIENTIFIC RIGOUR

This Declaration acknowledges that this paper adheres to the principles for transparent reporting and scientific rigour of preclinical research as stated in the *BJP* guidelines for https://bpspubs.onlinelibrary.wiley.com/doi/full/10.1111/bph.14207, and https://bpspubs.onlinelibrary.wiley.com/doi/full/10.1111/bph.14206, and as recommended by funding agencies, publishers and other organisations engaged with supporting research.

## Supporting information

Table S1. Reference tracers (concentration, nM), and reference competitors used for radioligand binding competition assays for the different receptors studied. *Historical pIC50 values obtained at Ogeda S.A. (now Epics Therapeutics S.A., Gosselies, Belgium). Values represent mean values ± SEM of a stated number of averaged technical duplicates (n).
Table S2. Reference agonists for second messenger activation assays of cAMP (5‐HT1A/B/E/F and 5‐HT7), GTPγS (5‐HT1D) and IP (5‐HT2A/B). *Historical pEC50 values obtained at Ogeda S.A. (now Epics Therapeutics S.A., Gosselies, Belgium). Values represent mean values ± SEM of a stated number of averaged technical duplicates (n).
Table S3. Summary of pEC_50_ values of vasoconstriction of the human coronary artery. These values represent the negative logarithm of the molar concentration of these compounds at which 50% of their maximal response was exerted. When a compound was devoid of vasoconstrictor activity, a pEC_50_ of 5 was set.
Table S4. Comparison pIC50 (negative logarithm of the molar concentration of these compounds at which 50% of the radioligand is displaced) and pKi (negative logarithm of the molar concentration of the dissociation constant) values of individual antimigraine drugs at 5‐HT1A/B/D/E/F, 5‐HT2A/B and 5‐HT7 receptors obtained in our study and values reported in the literature (Lit.) obtained from Adham et al., 1993a; Adham et al., 1993b; Bard, Kucharewicz, Zgombick, Weinshank, Branchek & Cohen, 1996; Barf et al., 1996; Beer, Heald, McAllister & Stanton, 1998; Bhalla, Sharma, Ma, Wurch, Pauwels & Saxena, 2001; Bou et al., 2000; Brüss, Kiel, Bönisch, Kostanian & Göthert, 2005; Castro et al., 1997; Choi et al., 2008; Comer & Hons, 2002; Connor et al., 1997; Deleu & Hanssens, 2000; Dickenson & Hill, 1998; Dupuis, Perez, Halazy, Colpaert & Pauwels, 1999; Ghoneim, Ibrahim, El‐Deeb, Lee & Booth, 2011; Glennon et al., 2000; Goadsby, 1998; Gras, Llenas, Jansat, Jáuregui, Cabarrocas & Palacios, 2002; Hoyer, 1988; John et al., 1999; Johnson et al., 1997; Knight et al., 2004; Leonhardt, Herrick‐Davis & Titeler, 1989; Leysen et al., 1996; Lovenberg et al., 1993; Martin et al., 1997; McAllister et al., 1992; Napier, Stewart, Melrose, Hopkins, McHarg & Wallis, 1999; Nelson et al., 2010; Newman‐Tancredi et al., 1997; Pauwels, Palmier, Dupuis & Colpaert, 1998; Pauwels, Palmier, Wurch & Colpaert, 1996; Pauwels, Tardif, Palmier, Wurch & Colpaert, 1997; Phebus et al., 1997; Razzaque et al., 1999; Schmuck, Ullmer, Kalkman, Probst & Lübbert, 1996; Shen, Monsma, Metcalf, Jose, Hamblin & Sibley, 1993; Stanton & Beer, 1997; Sternfeld et al., 1999; Street et al., 1995; Vries, Villalón & Saxena, 1999; Wang et al., 2013; Wurch, Palmier & Pauwels, 2000; Xu et al., 1999; Zgombick, Schechter, Macchi, Hartig, Branchek & Weinshank, 1992; Zgombick, Weinshank, Macchi, Schechter, Branchek & Hartig, 1991; Zhang et al., 2004.
Table S5. Comparison of pEC50 values of cAMP (5‐HT1A/B/E/F and 5‐HT7), GTPγS (5‐HT1A/B/D/E/F) and IP (5‐HT2) assays of individual antimigraine drugs obtained in our study and the historical values reported in the literature (Lit.). These values represent the negative logarithm of the molar concentration of these compounds at which 50% of their maximal response is exerted. The lesser than 5 symbol (<5) indicates that less than 50% response was obtained at 10 μM. Historical values were obtained from Beer, Heald, McAllister & Stanton, 1998; Bhalla, Sharma, Ma, Wurch, Pauwels & Saxena, 2001; Bou et al., 2000; Castro et al., 1997; Dupuis, Perez, Halazy, Colpaert & Pauwels, 1999; John et al., 1999; Johnson et al., 1997; Nelson et al., 2010; Newman‐Tancredi et al., 1997; Pauwels, Palmier, Dupuis & Colpaert, 1998; Pauwels, Tardif, Palmier, Wurch & Colpaert, 1997; Razzaque et al., 1999; Schmuck, Ullmer, Kalkman, Probst & Lübbert, 1996; Stanton & Beer, 1997; Sternfeld et al., 1999; Wang et al., 2013 and Xu et al., 1999.
Figure S1. Correlation between the pKi values obtained from literature and the pKi values obtained in our study for lasmiditan, triptans (sumatriptan, zolmitriptan, naratriptan, rizatriptan, almotriptan, eletriptan, frovatriptan, donitriptan, avitriptan) and other 5‐HT receptors ligands (ergotamine, alniditan, 5HT, 5‐carboxamidotryptamine). For references see Table S5.
Figure S2. Functional responses (cAMP assay) to 5‐CT in CHO cells transfected with 5‐HT1B receptor (upper) and in CHO cells transfected with an unrelated G protein‐coupled receptor.
Figure S3. Correlation between second messenger activation (*i.e.* cAMP, IP) and the contractile potency of lasmiditan, triptans (sumatriptan, zolmitriptan, naratriptan, rizatriptan, almotriptan, eletriptan, frovatriptan, donitriptan, avitriptan) and other 5‐HT receptors ligands (ergotamine, 5HT, 5‐CT) in human isolated coronary arteries; N.S., non‐significant; *P < 0.05.
Figure S4. Correlation between the second messenger activation of 5‐HT1B receptor *vs* 5‐HT1F receptor by lasmiditan, triptans (sumatriptan, zolmitriptan, naratriptan, rizatriptan, almotriptan, eletriptan, frovatriptan, donitriptan, avitriptan) and other 5‐HT receptors ligands (ergotamine, 5HT, 5‐carboxamidotryptamine) in human isolated coronary arteries; *P < 0.05.
