# Supplementary material for: Characterization of binding, functional activity, and contractile responses of the selective 5‐HT1F receptor agonist lasmiditan
Source: Br J Pharmacol. 2019 Nov 7;176(24):4681–95. doi: 10.1111/bph.14832 (PMC6965684; doi:10.1111/bph.14832)
Supplement: Supplementary file 1 — Table S1. Reference tracers (concentration, nM), and reference competitors used for radioligand binding competition assays for the different receptors studied. *Historical pIC50 values obtained at Ogeda S.A. (now Epics Therapeutics S.A., Gosselies, Belgium). Values represent mean values ± SEM of a stated number of averaged technical duplicates (n). Table S2. Reference agonists for second messenger activation assays of cAMP (5‐HT1A/B/E/F and 5‐HT7), GTPγS (5‐HT1D) and IP (5‐HT2A/B). *Historical pEC50 values obtained at Ogeda S.A. (now Epics Therapeutics S.A., Gosselies, Belgium). Values represent mean values ± SEM of a stated number of averaged technical duplicates (n). Table S3. Summary of pEC50 values of vasoconstriction of the human coronary artery. These values represent the negative logarithm of the molar concentration of these compounds at which 50% of their maximal response was exerted. When a compound was devoid of vasoconstrictor activity, a pEC50 of 5 was set. Table S4. Comparison pIC50 (negative logarithm of the molar concentration of these compounds at which 50% of the radioligand is displaced) and pKi (negative logarithm of the molar concentration of the dissociation constant) values of individual antimigraine drugs at 5‐HT1A/B/D/E/F, 5‐HT2A/B and 5‐HT7 receptors obtained in our study and values reported in the literature (Lit.) obtained from Adham et al., 1993a; Adham et al., 1993b; Bard, Kucharewicz, Zgombick, Weinshank, Branchek & Cohen, 1996; Barf et al., 1996; Beer, Heald, McAllister & Stanton, 1998; Bhalla, Sharma, Ma, Wurch, Pauwels & Saxena, 2001; Bou et al., 2000; Brüss, Kiel, Bönisch, Kostanian & Göthert, 2005; Castro et al., 1997; Choi et al., 2008; Comer & Hons, 2002; Connor et al., 1997; Deleu & Hanssens, 2000; Dickenson & Hill, 1998; Dupuis, Perez, Halazy, Colpaert & Pauwels, 1999; Ghoneim, Ibrahim, El‐Deeb, Lee & Booth, 2011; Glennon et al., 2000; Goadsby, 1998; Gras, Llenas, Jansat, Jáuregui, Cabarrocas & Palacios, 2002; Hoyer, 1988; John [file BPH-176-4681-s001.pdf]

Supplementary material

Supplementary Table 1. Reference tracers (concentration, nM), and reference competitors used for radioligand binding competition assays for the different receptors studied. *Historical pIC_{50} values obtained at Ogeda S.A. (now Epics Therapeutics S.A., Gosselies, Belgium). Values represent mean values ± SEM of a stated number of averaged technical duplicates (n).

Receptor	Reference tracer	Assay concentration (nM)	Reference competitor	Historical* pIC_{50} (reference competitor)	Estimated pIC_{50} (reference competitor)	pK_i (reference competitor)
$5\text{-}HT_{1A}$	$[^3H]$-8-OH-DPAT	0.39	5-HT hydrochloride	8.83 ± 0.04 (23)	9.00 ± 0.06 (2)	9.51 ± 0.06 (2)
$5\text{-}HT_{1B}$	$[^3H]$-5-CT	0.60	5-HT hydrochloride	8.42 ± 0.10 (8)	8.61 ± 0.19 (2)	9.09 ± 0.19 (2)
$5\text{-}HT_{1D}$	$[^3H]$-5-CT	0.50	5-HT hydrochloride	8.35 ± 0.08 (7)	8.67 ± 0.18 (2)	9.35 ± 0.18 (2)
$5\text{-}ht_{1E}$	$[^3H]$-LSD	14.0	BRL-54443	8.49 ± 0.09 (5)	8.44 ± 0.07 (3)	8.74 ± 0.07 (3)
$5\text{-}HT_{1F}$	$[^3H]$-LSD	8.00	BRL-54443	8.59 ± 0.17 (5)	8.54 ± 0.12 (3)	8.96 ± 0.12 (3)
$5\text{-}HT_{2A}$	$[^3H]$-Ketanserin	1.48	Ketanserin	8.22 ± 0.18 (6)	8.17 ± 0.04 (2)	8.69 ± 0.04 (2)
$5\text{-}HT_{2B}$	$[^3H]$-Mesulergin	1.00	5-HT hydrochloride	7.67 ± 0.09 (8)	7.68 ± 0.05 (3)	7.89 ± 0.05 (3)
$5\text{-}HT_7$	$[^3H]$-LSD	1.00	5-CT maleate	9.28 ± 0.05 (13)	9.42 ± 0.17 (3)	9.51 ± 0.17 (3)

Supplementary Table 2. Reference agonists for second messenger activation assays of cAMP ($5\text{-}HT_{1A/B/E/F}$ and $5\text{-}HT_7$), GTPγS ($5\text{-}HT_{1D}$) and IP ($5\text{-}HT_{2A/B}$). *Historical pEC_{50} values obtained at Ogeda S.A. (now Epics Therapeutics S.A., Gosselies, Belgium). Values represent mean values ± SEM of a stated number of averaged technical duplicates (n).

Receptor	Reference agonist	Historical* pEC_{50} cAMP/IP/ GTPγS	Estimated pEC_{50} cAMP/IP/ GTPγS
$5\text{-}HT_{1A}$	5-CT maleate	9.18 ± 0.05 (35)	9.02 ± 0.17 (4)
$5\text{-}HT_{1B}$	5-CT maleate	8.78 ± 0.07 (23)	8.80 ± 0.01 (2)
$5\text{-}HT_{1D}$	5-CT maleate	9.30 ± 0.05 (21)	9.26 ± 0.13 (3)
$5\text{-}ht_{1E}$	5-HT hydrochloride	9.00 ± 0.16 (2)	8.61 ± 0.05 (3)
$5\text{-}HT_{1F}$	5-HT hydrochloride	8.94 ± 0.15 (7)	8.69 ± 0.16 (3)
$5\text{-}HT_{2A}$	α-Me-5-HT	8.68 ± 0.05 (31)	8.33 ± 0.06 (2)
$5\text{-}HT_{2B}$	α-Me-5-HT	9.70 ± 0.09 (28)	9.63 ± 0.08 (2)
$5\text{-}HT_7$	5-CT maleate	9.59 ± 0.03 (45)	9.62 ± 0.01 (3)

Supplementary Table 3. Summary of pEC_{50} values of vasoconstriction of the human coronary artery. These values represent the negative logarithm of the molar concentration of these compounds at which 50% of their maximal response was exerted. When a compound was devoid of vasoconstrictor activity, a pEC_{50} of 5 was set.

Agonist	pEC_{50}	Reference
5-HT hydrochloride	6.50	(MaassenVanDenBrink, Reekers, Bax, Ferrari & Saxena, 1998; Parsons et al., 1998)
5-CT maleate	6.44	(MaassenVanDenBrink, Reekers, Bax & Saxena, 2000)
Ergotamine tartrate	7.81	(MaassenVanDenBrink, Reekers, Bax, Ferrari & Saxena, 1998)
Sumatriptan succinate	6.11	(MaassenVanDenBrink, Reekers, Bax, Ferrari & Saxena, 1998)
Zolmitriptan	6.33	(MaassenVanDenBrink, Reekers, Bax, Ferrari & Saxena, 1998)
Naratriptan hydrochloride	6.78	(MaassenVanDenBrink, Reekers, Bax, Ferrari & Saxena, 1998)
Rizatriptan benzoate	6.36	(MaassenVanDenBrink, Reekers, Bax, Ferrari & Saxena, 1998)
Eletriptan hydrobromide	5.54	(van den Broek et al., 2000)
Frovatriptan Racemate	7.86	(Parsons et al., 1998)
Donitriptan hydrochloride	8.25	(van den Broek et al., 2002)
Avitriptan fumarate	7.06	(MaassenVanDenBrink, Reekers, Bax, Ferrari & Saxena, 1998; Saxena et al., 1997)
Lasmiditan hemisuccinate	5.00	

Supplementary Table 4. Comparison pIC_{50} (negative logarithm of the molar concentration of these compounds at which 50% of the radioligand is displaced) and pK_i (negative logarithm of the molar concentration of the dissociation constant) values of individual antimigraine drugs at $5\text{-}HT_{1A/B/D/E/F}$, $5\text{-}HT_{2A/B}$ and $5\text{-}HT_7$ receptors obtained in our study and values reported in the literature (Lit.) obtained from Adham et al., 1993a; Adham et al., 1993b; Bard, Kucharewicz, Zgombick, Weinshank, Branchek & Cohen, 1996; Barf et al., 1996; Beer, Heald, McAllister & Stanton, 1998; Bhalla, Sharma, Ma, Wurch, Pauwels & Saxena, 2001; Bou et al., 2000; Brüss, Kiel, Bönisch, Kostanian & Göthert, 2005; Castro et al., 1997; Choi et al., 2008; Comer & Hons, 2002; Connor et al., 1997; Deleu & Hanssens, 2000; Dickenson & Hill, 1998; Dupuis, Perez, Halazy, Colpaert & Pauwels, 1999; Ghoneim, Ibrahim, El-Deeb, Lee & Booth, 2011; Glennon et al., 2000; Goadsby, 1998; Gras, Llenas, Jansat, Jáuregui, Cabarrocas & Palacios, 2002; Hoyer, 1988; John et al., 1999; Johnson et al., 1997; Knight et al., 2004; Leonhardt, Herrick-Davis & Titeler, 1989; Leysen et al., 1996; Lovenberg et al., 1993; Martin et al., 1997; McAllister et al., 1992; Napier, Stewart, Melrose, Hopkins, McHarg & Wallis, 1999; Nelson et al., 2010; Newman-Tancredi et al., 1997; Pauwels, Palmier, Dupuis & Colpaert, 1998; Pauwels, Palmier, Wurch & Colpaert, 1996; Pauwels, Tardif, Palmier, Wurch & Colpaert, 1997; Phebus et al., 1997; Razzaque et al., 1999; Schmuck, Ullmer, Kalkman, Probst & Lübbert, 1996; Shen, Monsma, Metcalf, Jose, Hamblin & Sibley, 1993; Stanton & Beer, 1997; Sternfeld et al., 1999; Street et al., 1995; Vries, Villalón & Saxena, 1999; Wang et al., 2013; Wurch, Palmier & Pauwels, 2000; Xu et al., 1999; Zgombick, Schechter, Macchi, Hartig, Branchek & Weinshank, 1992; Zgombick, Weinshank, Macchi, Schechter, Branchek & Hartig, 1991; Zhang et al., 2004.

Agonist	$5\text{-}HT_{1A}$				$5\text{-}HT_{1B}$				$5\text{-}HT_{1D}$				$5\text{-}ht_{1E}$				$5\text{-}HT_{1F}$				$5\text{-}HT_{2A}$				$5\text{-}HT_{2B}$				$5\text{-}HT_7$			
	pIC_{50}		pK_i		pIC_{50}		pK_i		pIC_{50}		pK_i		pIC_{50}		pK_i		pIC_{50}		pK_i		pIC_{50}		pK_i		pIC_{50}		pK_i		pIC_{50}		pK_i	
	Our	Lit.	Our	Lit.	Our	Lit.	Our	Lit.	Our	Lit.	Our	Lit.	Our	Lit.	Our	Lit.	Our	Lit.	Our	Lit.	Our	Lit.	Our	Lit.	Our	Lit.	Our	Lit.	Our	Lit.	Our	Lit.
Ergotamine tartrate	9.19	9.11	9.70	9.17	8.87	-	9.34	8.45	8.63	-	9.31	9.20	6.08	-	6.39	7.07	6.71	-	7.13	6.78	7.62	8.19	8.14	9.01	7.73	8.46	7.94	8.71	7.13	7.49	7.23	7.49
Sumatriptan succinate	6.63	6.32	7.14	6.53	7.81	8.12	8.29	7.92	8.31	7.98	9.00	8.32	5.42	5.40	5.72	5.83	7.13	7.70	7.55	7.67	<5	<5	<5	<5	<5	<5	<5	<5	6.10	5.44	6.19	6.23
Zolmitriptan	7.28	6.50	7.79	7.26	8.85	8.21	9.33	8.33	9.28	8.75	9.97	9.19	7.51	-	7.81	7.84	7.13	7.27	7.55	7.39	<5	<5	<5	<5	<5	<5	<5	-	6.97	-	7.06	7.01
Naratriptan hydrochloride	7.31	6.90	7.82	7.29	8.75	-	9.22	8.69	8.62	-	9.30	8.83	7.83	-	8.13	7.61	8.33	-	8.75	8.31	<5	-	<5	<5	<5	-	5.08	-	5.84	-	5.93	5.5
Rizatriptan benzoate	6.81	6.50	7.32	6.48	7.51	7.39	7.99	7.63	8.15	7.63	8.83	8.17	6.48	-	6.78	6.78	6.40	-	6.82	6.71	<5	5.20	<5	<5	5.30	-	5.51	6.59	<5	-	<5	5.73
Almotriptan malate	6.23	6.07	6.73	6.30	7.97	7.92	8.45	7.60	7.57	7.89	8.26	7.90	<5	-	<5	-	7.15	7.30	7.57	7.10	<5	<5	<5	<5	<5	<5	<5	<5	6.36	5.50	6.46	6.5
Eletriptan hydrobromide	8.20	-	8.71	7.38	8.80	-	9.28	8.00	9.31	-	9.99	8.92	6.91	-	7.21	7.33	7.35	-	7.77	8.01	5.42	-	5.94	<5.5	6.14	-	6.35	-	6.61	-	6.70	6.7
Frovatriptan racemate	6.83	-	7.34	7.26	8.09	-	8.57	8.40	8.10	-	8.78	8.39	<5	-	5.18	-	6.50	-	6.92	7.00	<5	-	<5	5.30	<5	-	<5	-	6.88	-	6.97	6.70
Donitriptan hydrochloride	7.42	-	7.93	7.60	9.29	-	9.77	9.75	9.18	-	9.86	9.70	5.47	-	5.77	5.94	<5	-	5.18	5.45	5.83	-	6.35	6.81	5.88	-	6.09	-	6.12	-	6.21	6.43
Avitriptan fumarate	7.20	-	7.71	7.10	8.32	-	8.80	7.69	8.42	-	9.11	8.33	5.15	-	5.45	6.00	6.69	-	7.11	6.75	5.11	-	5.63	-	5.73	-	5.94	-	6.03	-	6.12	-
Alniditan dihydrochloride	8.81	8.32	9.32	8.47	8.93	9.03	9.41	9.40	8.66	8.65	9.35	8.96	5.98	6.2	6.28	6.62	6.02	6.2	6.44	6.24	<5	<5	5.43	<5	6.67	-	6.88	-	7.16	6.5	7.26	6.59
Lasmiditan hemisuccinate	5.88	-	6.39	5.98	5.54	-	6.02	5.98	5.62	-	6.31	5.87	5.54	-	5.84	6.23	8.09	-	8.51	8.66	<5	-	<5	<5	5.01	-	5.22	<5	<5	-	<5	<5
LY334370 hydrochloride	7.98	-	8.49	7.72	6.74	-	7.21	6.82	6.24	-	6.92	6.62	6.83	-	7.13	6.75	9.03	-	9.45	8.73	5.11	-	5.63	5.82	5.98	-	6.19	5.89	5.66	-	5.75	5.81
LY344864 hydrochloride	6.12	-	6.63	6.27	6.13	-	6.61	6.26	5.83	-	6.52	6.24	6.05	-	6.35	5.85	8.38	-	8.80	8.22	5.11	-	5.63	5.41	5.31	-	5.52	5.77	5.69	-	5.78	5.31

Supplementary Table 5. Comparison of pEC_{50} values of cAMP (5-$HT_{1A/B/E/F}$ and 5-HT_7), GTPγS (5-$HT_{1A/B/D/E/F}$) and IP (5-HT_2) assays of individual antimigraine drugs obtained in our study and the historical values reported in the literature (Lit.). These values represent the negative logarithm of the molar concentration of these compounds at which 50% of their maximal response is exerted. The lesser than 5 symbol (<5) indicates that less than 50% response was obtained at 10 µM. Historical values were obtained from Beer, Heald, McAllister & Stanton, 1998; Bhalla, Sharma, Ma, Wurch, Pauwels & Saxena, 2001; Bou et al., 2000; Castro et al., 1997; Dupuis, Perez, Halazy, Colpaert & Pauwels, 1999; John et al., 1999; Johnson et al., 1997; Nelson et al., 2010; Newman-Tancredi et al., 1997; Pauwels, Palmier, Dupuis & Colpaert, 1998; Pauwels, Tardif, Palmier, Wurch & Colpaert, 1997; Razzaque et al., 1999; Schmuck, Ullmer, Kalkman, Probst & Lübbert, 1996; Stanton & Beer, 1997; Sternfeld et al., 1999; Wang et al., 2013 and Xu et al., 1999.

Agonist	5-HT_{1A}				5-HT_{1B}				5-HT_{1D}		5-ht_{1E}				5-HT_{1F}				5-HT_{2A}		5-HT_{2B}		5-HT_7	
	pEC_{50} cAMP		pEC_{50} GTP		pEC_{50} cAMP		pEC_{50} GTP		pEC_{50} GTP		pEC_{50} cAMP		pEC_{50} GTP		pEC_{50} cAMP		pEC_{50} GTP		pEC_{50} IP		pEC_{50} IP		pEC_{50} cAMP	
	Our	Lit.	Our	Lit.	Our	Lit.	Our	Lit.	Our	Lit.	Our	Lit.	Our	Lit.	Our	Lit.	Our	Lit.	Our	Lit.	Our	Lit.	Our	Lit.
Ergotamine tartrate	9.78	-	9.63	-	9.94	9.58	9.52	-	9.43	-	5.95	-	5.74	-	5.97	-	6.30	-	9.25	-	8.72	-	7.09	-
Sumatriptan succinate	<5	<5	<5	5.34	7.32	7.73	7.91	6.87	8.30	7.76	5.99	-	5.79	5.00	8.03	7.46	6.80	6.61	<5	<5	<5	5.18	5.22	<5
Zolmitriptan	<5	-	5.52	5.65	7.87	7.89	8.42	7.43	9.51	8.91	8.18	-	7.81	7.21	8.00	8.15	6.67	6.38	<5	<5	<5	<5	6.28	5.30
Naratriptan hydrochloride	<5	-	6.52	5.78	8.05	7.805	8.86	7.81	8.80	8.46	7.75	-	8.17	7.50	8.38	8.66	8.05	7.71	<5	-	<5	-	<5	-
Rizatriptan benzoate	<5	-	<5	5.44	7.08	7.34	7.56	6.63	8.11	7.89	7.34	-	6.90	-	6.54	7.60	5.91	-	<5	-	5.49	5.65	<5	-
Almotriptan malate	<5	-	5.48	-	7.08	8.80	7.85	-	7.75	-	<5	-	<5	-	7.79	-	6.90	-	<5	5	5.20	<5	<5	-
Eletriptan hydrobromide	5.74	-	6.38	-	8.00	8.44	8.09	-	9.04	-	7.53	-	6.90	-	8.13	-	6.88	-	6.07	6.12	6.81	-	6.45	-
Frovatriptan racemate	<5	-	6.12	5.94	7.98	-	8.14	7.70	8.36	8.64	5.04	-	<5	<5	7.10	-	6.35	6.44	<5	-	<5	-	7.42	-
Donitriptan hydrochloride	5.94	-	6.74	-	9.96	9.51	9.52	8.74	9.51	9.08	<5	-	<5	-	<5	-	<5	-	8.10	-	7.61	6.74	5.23	-
Avitriptan fumarate	<5	-	6.19	-	8.57	-	8.68	-	9.27	-	5.52	-	<5	-	7.09	-	6.05	-	6.91	-	6.41	-	5.38	-
Alniditan dihydrochloride	7.00	-	7.29	6.94	8.87	-	8.90	-	8.20	-	5.68	-	5.21	-	5.92	-	5.17	-	<5	-	7.15	-	6.32	-
Lasmiditan hemisuccinate	<5	-	<5	<5	<5	-	<5	<5	6.64	<5	6.17	-	5.34	<5	8.43	-	7.80	7.37	<5	-	<5	-	<5	-
LY334370 hydrochloride	5.84	-	6.96	-	6.52	-	5.80	-	6.92	-	7.53	-	6.95	-	9.08	8.82	9.38	-	<5	-	<5	-	<5	-
LY344864 hydrochloride	<5	-	<5	-	<5	-	5.82	-	6.93	-	6.22	-	6.12	-	8.72	-	7.85	-	<5	-	<5	-	<5	<5

Supplementary Figure 1. Correlation between the pK_i values obtained from literature and the pK_i values obtained in our study for lasmiditan, triptans (sumatriptan, zolmitriptan, naratriptan, rizatriptan, almotriptan, eletriptan, frovatriptan, donitriptan, avitriptan) and other 5-HT receptors ligands (ergotamine, alniditan, 5-HT, 5-carboxamidotryptamine). For references see Supplementary Table 5.

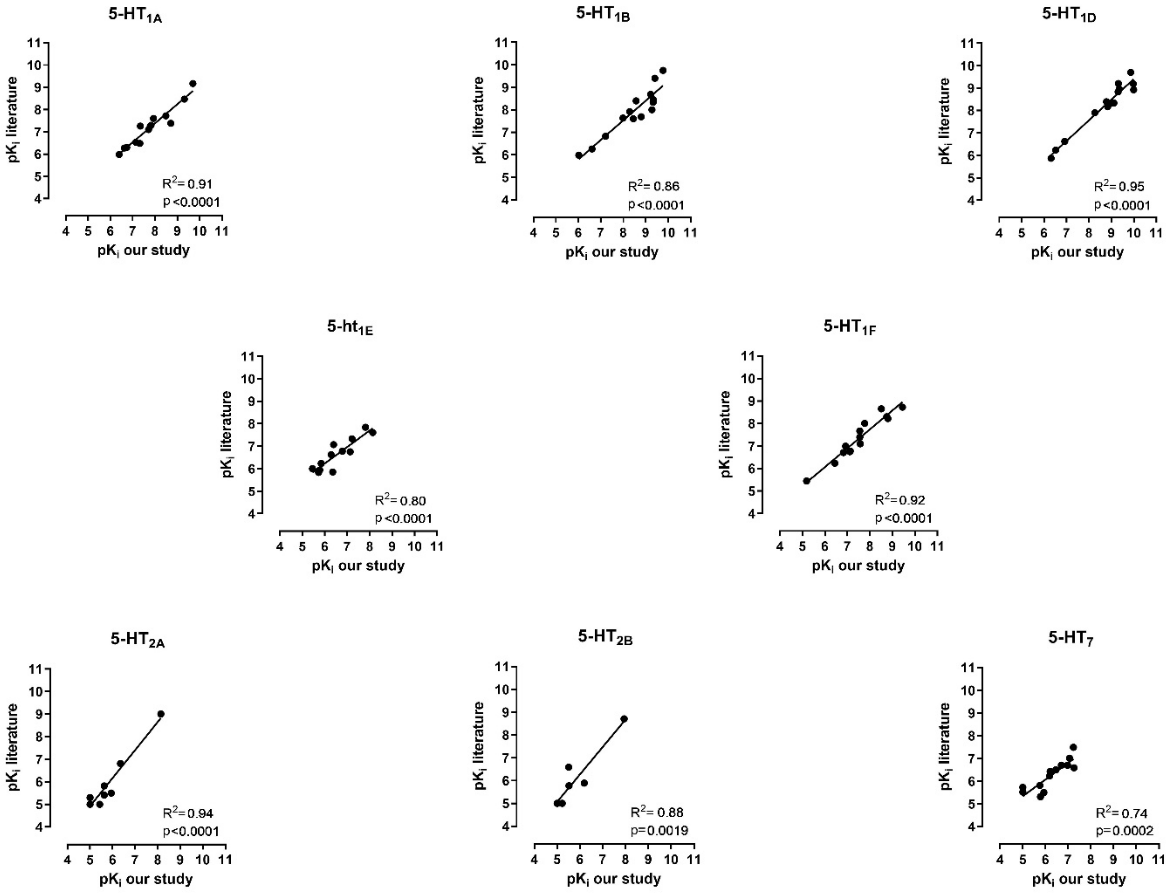

Supplementary Figure 2. Functional responses (cAMP assay) to 5-CT in CHO cells transfected with $5\text{-}HT_{1B}$ receptor (upper) and in CHO cells transfected with an unrelated G protein-coupled receptor.

$5\text{-}HT_{1B}$ receptor transfected CHO cells

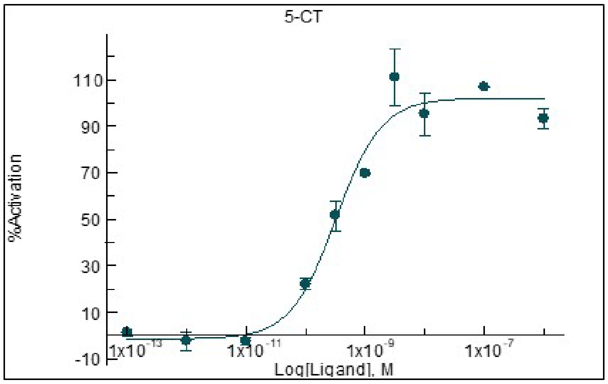

Non-$5\text{-}HT_{1B}$ receptor transfected CHO cells

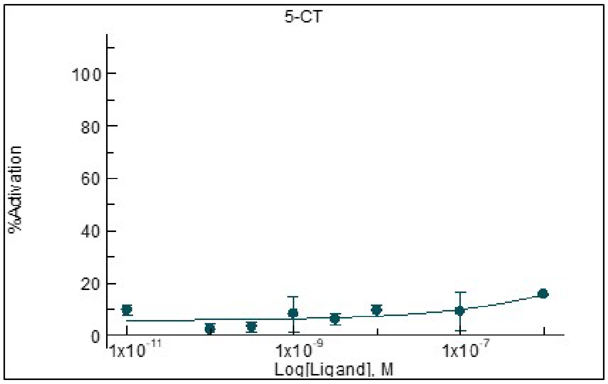

Supplementary Figure 3. Correlation between second messenger activation (*i.e.* cAMP, IP) and the contractile potency of lasmiditan, triptans (sumatriptan, zolmitriptan, naratriptan, rizatriptan, almotriptan, eletriptan, frovatriptan, donitriptan, avitriptan) and other 5-HT receptors ligands (ergotamine, 5-HT, 5-CT) in human isolated coronary arteries; N.S., non-significant; *P<0.05.

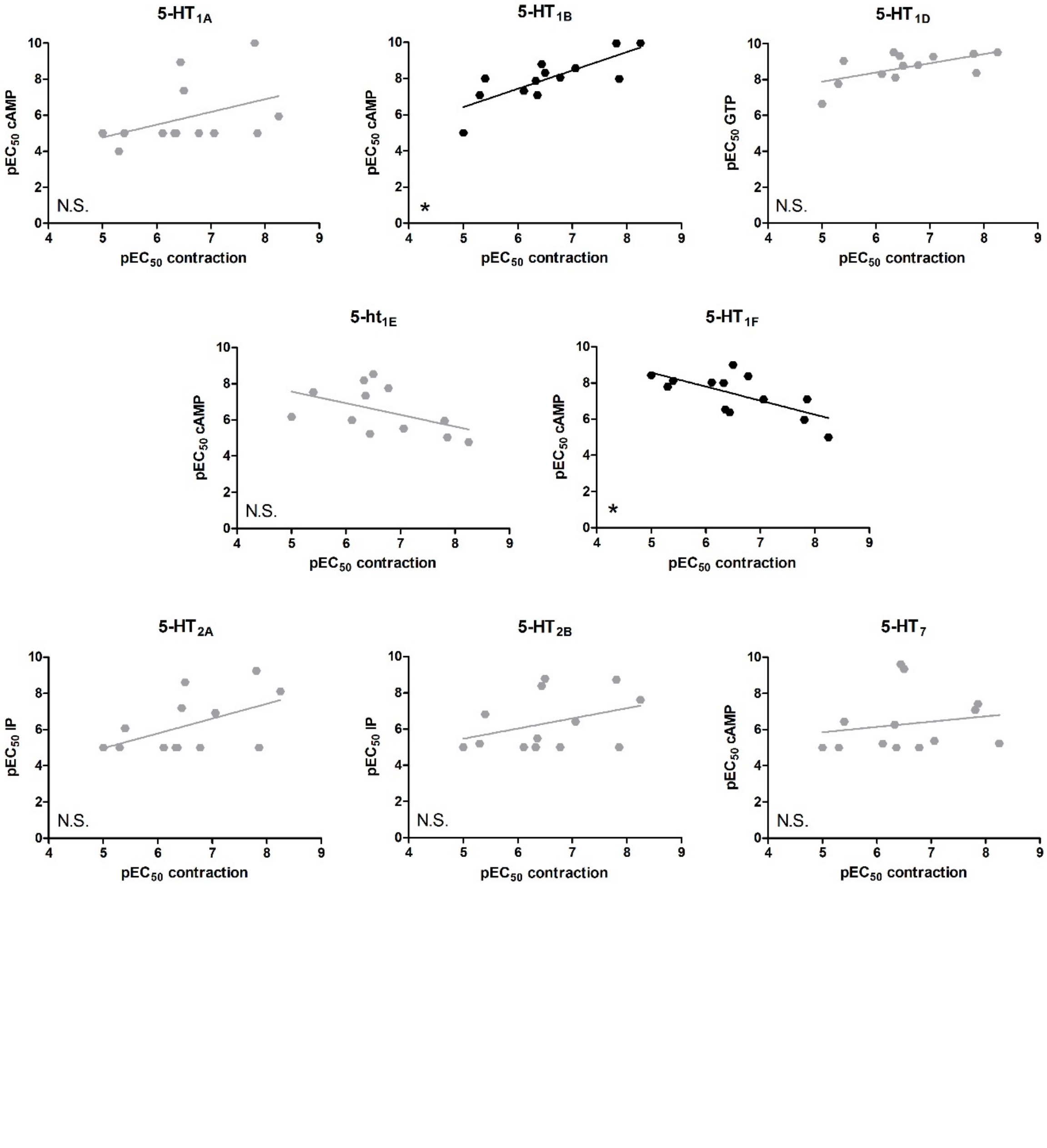

Supplementary Figure 4. Correlation between the second messenger activation of 5-HT_{1B} receptor *vs* 5-HT_{1F} receptor by lasmiditan, triptans (sumatriptan, zolmitriptan, naratriptan, rizatriptan, almotriptan, eletriptan, frovatriptan, donitriptan, avitriptan) and other 5-HT receptors ligands (ergotamine, 5-HT, 5-carboxamidotryptamine) in human isolated coronary arteries; *P<0.05.

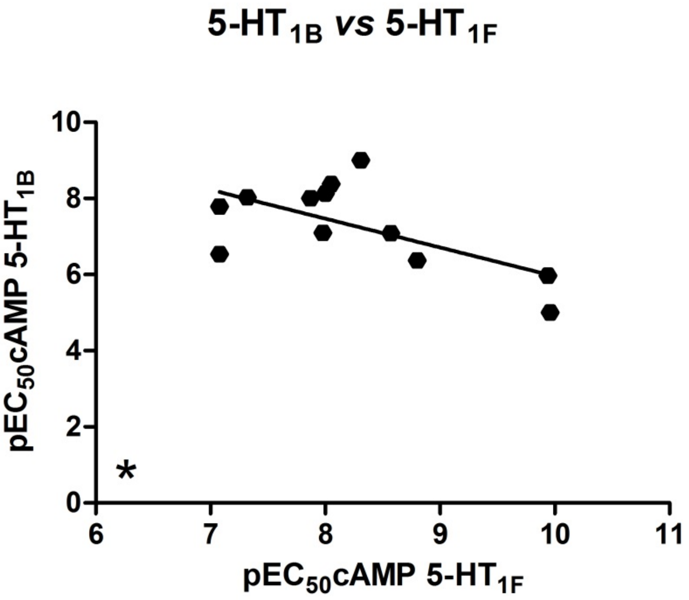

References

Adham N, Borden LA, Schechter LE, Gustafson EL, Cochran TL, Vaysse PJJ, et al. (1993a). Cell-specific coupling of the cloned human 5-HT1F receptor to multiple signal transduction pathways. Naunyn-Schmiedeberg's Archives of Pharmacology 348: 566-575.

Adham N, Kao HT, Schecter LE, Bard J, Olsen M, Urquhart D, et al. (1993b). Cloning of another human serotonin receptor (5-HT1F): a fifth 5-HT1 receptor subtype coupled to the inhibition of adenylate cyclase. Proceedings of the National Academy of Sciences of the United States of America 90: 408-412.

Bard JA, Kucharewicz SA, Zgombick JM, Weinshank RL, Branchek TA, & Cohen ML (1996). Differences in ligand binding profiles between cloned rabbit and human 5-HT1Dα and 5-HT1Dβ receptors: ketanserin and methiothepin distinguish rabbit 5-HT1D receptor subtypes. Naunyn-Schmiedeberg's Archives of Pharmacology 354: 237-244.

Barf TA, de Boer P, Wikström H, Peroutka SJ, Svensson K, Ennis MD, et al. (1996). 5-HT1D Receptor Agonist Properties of Novel 2-[5-[[(Trifluoromethyl)sulfonyl]oxy]indolyl]ethylamines and Their Use as Synthetic Intermediates. Journal of Medicinal Chemistry 39: 4717-4726.

Beer MS, Heald MA, McAllister G, & Stanton JA (1998). Pharmacological characterisation of a cloned dog 5-HT1B receptor cell line. European Journal of Pharmacology 360: 117-121.

Bhalla P, Sharma HS, Ma X, Wurch T, Pauwels PJ, & Saxena PR (2001). Molecular cloning, pharmacological properties and tissue distribution of the porcine 5-HT1B receptor. British Journal of Pharmacology 133: 891-901.

Bou J, Domènech T, Puig J, Heredia A, Gras J, Fernández-Forner D, et al. (2000). Pharmacological characterization of almotriptan: an indolic 5-HT receptor agonist for the treatment of migraine. European Journal of Pharmacology 410: 33-41.

van den Broek RWM, MaassenVanDenBrink A, de Vries R, Bogers AJJC, Stegmann APA, Avezaat CJ, et al. (2000). Pharmacological analysis of contractile effects of eletriptan and sumatriptan on human isolated blood vessels. European Journal of Pharmacology 407: 165-173.

van den Broek RWM, MaassenVanDenBrink A, Mulder PGH, Bogers AJJC, Avezaat CJJ, John GW, et al. (2002). Comparison of contractile responses to donitriptan and sumatriptan in the human middle meningeal and coronary arteries. European Journal of Pharmacology 443: 125-132.

Brüss M, Kiel S, Bönisch H, Kostanian A, & Göthert M (2005). Decreased agonist, but not antagonist, binding to the naturally occurring Thr92Lys variant of the h5-HT7(a) receptor. Neurochemistry International 47: 196-203.

Castro JL, Street LJ, Guiblin AR, Jelley RA, Russell MGN, Sternfeld F, et al. (1997). 3-[2-(Pyrrolidin-1-yl)ethyl]indoles and 3-[3-(Piperidin-1-yl)propyl]indoles: Agonists for the h5-HT1D Receptor with High Selectivity over the h5-HT1B Subtype. Journal of Medicinal Chemistry 40: 3497-3500.

Choi S-K, Green D, Ho A, Klein U, Marquess D, Taylor R, et al. (2008). Designing Selective, High Affinity Ligands of 5-HT1D Receptor by Covalent Dimerization of 5-HT1F Ligands Derived From 4-Fluoro-N-[3-(1-methyl-4-piperidinyl)-1H-indol-5-yl]benzamide. Journal of Medicinal Chemistry 51: 3609-3616.

Comer MB, & Hons (2002). Pharmacology of the Selective 5-HT1B/1D Agonist Frovatriptan. Headache: The Journal of Head and Face Pain 42: 47-53.

Connor HE, Feniuk W, Beattie DT, North PC, Oxford AW, Saynor DA, et al. (1997). Naratriptan:Biological Profile in Animal Models Relevant to Migraine. Cephalalgia 17: 145-152.

Deleu D, & Hanssens Y (2000). Current and Emerging Second-Generation Triptans in Acute Migraine Therapy: A Comparative Review. The Journal of Clinical Pharmacology 40: 687-700.

Dickenson JM, & Hill SJ (1998). Human 5-HT1B receptor stimulated inositol phospholipid hydrolysis in CHO cells: synergy with Gq-coupled receptors. European Journal of Pharmacology 348: 279-285.

Dupuis DS, Perez M, Halazy S, Colpaert FC, & Pauwels PJ (1999). Magnitude of 5-HT1B and 5-HT1A receptor activation in guinea-pig and rat brain: evidence from sumatriptan dimer-mediated [35S]GTPγS binding responses. Molecular Brain Research 67: 107-123.

Ghoneim OM, Ibrahim DA, El-Deeb IM, Lee SH, & Booth RG (2011). A novel potential therapeutic avenue for autism: design, synthesis and pharmacophore generation of SSRIs with dual action. Bioorganic & medicinal chemistry letters 21: 6714-6723.

Glennon RA, Lee M, Rangisetty JB, Dukat M, Roth BL, Savage JE, et al. (2000). 2-Substituted Tryptamines: Agents with Selectivity for 5-HT6 Serotonin Receptors. Journal of Medicinal Chemistry 43: 1011-1018.

Goadsby PJ (1998). Serotonin 5-HT1B/1D Receptor Agonists in Migraine. CNS Drugs 10: 271-286.

Gras J, Llenas J, Jansat JM, Jáuregui J, Cabarrocas X, & Palacios JM (2002). Almotriptan, a New Anti-Migraine Agent: A Review. CNS Drug Reviews 8: 217-234.

Hoyer D (1988). Functional Correlates of Serotonin 5-HT1 Recognition Sites. Journal of Receptor Research 8: 59-81.

John GW, Pauwels PJ, Perez M, Halazy S, Le Grand B, Verscheure Y, et al. (1999). F 11356, a Novel 5-Hydroxytryptamine (5-HT) Derivative with Potent, Selective, and Unique High Intrinsic Activity at 5-HT_{1B/1D} Receptors in Models Relevant to Migraine. Journal of Pharmacology and Experimental Therapeutics 290: 83-95.

Johnson KW, Schaus JM, Durkin MM, Audia JE, Kaldor SW, Flaugh ME, et al. (1997). 5-HT1F receptor agonists inhibit neurogenic dural inflammation in guinea pigs. NeuroReport 8: 2237-2239.

Knight AR, Misra A, Quirk K, Benwell K, Revell D, Kennett G, et al. (2004). Pharmacological characterisation of the agonist radioligand binding site of 5-HT2A, 5-HT2B and 5-HT2C receptors. Naunyn-Schmiedeberg's Archives of Pharmacology 370: 114-123.

Leonhardt S, Herrick-Davis K, & Titeler M (1989). Detection of a Novel Serotonin Receptor Subtype (5-HT1E) in Human Brain: Interaction with a GTP-Binding Protein. Journal of Neurochemistry 53: 465-471.

Leysen JE, Gommeren W, Heylen L, Luyten WH, Van de Weyer I, Vanhoenacker P, et al. (1996). Alniditan, a new 5-hydroxytryptamine1D agonist and migraine-abortive agent: ligand-binding properties of human 5-hydroxytryptamine1D alpha, human 5-hydroxytryptamine1D beta, and calf 5-

hydroxytryptamine1D receptors investigated with [3H]5-hydroxytryptamine and [3H]alniditan. Molecular Pharmacology 50: 1567-1580.

Lovenberg TW, Baron BM, de Lecea L, Miller JD, Prosser RA, Rea MA, et al. (1993). A novel adenylyl cyclase-activating serotonin receptor (5-HT7) implicated in the regulation of mammalian circadian rhythms. Neuron 11: 449-458.

MaassenVanDenBrink A, Reekers M, Bax WA, Ferrari MD, & Saxena PR (1998). Coronary Side-Effect Potential of Current and Prospective Antimigraine Drugs. Circulation 98: 25-30.

MaassenVanDenBrink A, Reekers M, Bax WA, & Saxena PR (2000). Human Isolated Coronary Artery Contraction to Sumatriptan Characterised by the Selective 5-HT1B/1D Receptor Antagonist GR55562. Pharmacology & Toxicology 86: 287-290.

Martin GR, Robertson AD, MacLennan SJ, Prentice DJ, Barrett VJ, Buckingham J, et al. (1997). Receptor specificity and trigemino-vascular inhibitory actions of a novel 5-HT1B/1D receptor partial agonist, 311C90 (zolmitriptan). British Journal of Pharmacology 121: 157-164.

McAllister G, Charlesworth A, Snodin C, Beer MS, Noble AJ, Middlemiss DN, et al. (1992). Molecular cloning of a serotonin receptor from human brain (5HT1E): a fifth 5HT1-like subtype. Proceedings of the National Academy of Sciences 89: 5517-5521.

Napier C, Stewart M, Melrose H, Hopkins B, McHarg A, & Wallis R (1999). Characterisation of the 5-HT receptor binding profile of eletriptan and kinetics of [3H]eletriptan binding at human 5-HT1B and 5-HT1D receptors. European Journal of Pharmacology 368: 259-268.

Nelson DL, Phebus LA, Johnson KW, Wainscott DB, Cohen ML, Calligaro DO, et al. (2010). Preclinical pharmacological profile of the selective 5-HT1F receptor agonist lasmiditan. Cephalalgia 30: 1159-1169.

Newman-Tancredi A, Conte C, Chaput C, Verrièle L, Audinot-Bouchez V, Lochon S, et al. (1997). Agonist activity of antimigraine drugs at recombinant human 5-HT1A receptors: potential implications for prophylactic and acute therapy. Naunyn-Schmiedeberg's Archives of Pharmacology 355: 682-688.

Parsons AA, Raval P, Smith S, Tilford N, King FD, Kaumann AJ, et al. (1998). Effects of the Novel High-Affinity 5-HT1B/1D-Receptor Ligand Frovatriptan in Human Isolated Basilar and Coronary Arteries. Journal of Cardiovascular Pharmacology 32: 220-224.

Pauwels PJ, Palmier C, Dupuis DS, & Colpaert FC (1998). Interaction of 5-HT1B/D ligands with recombinant h 5-HT1A receptors:Intrinsic activity and modulation by G-protein activation state. Naunyn-Schmiedeberg's Archives of Pharmacology 357: 490-499.

Pauwels PJ, Palmier C, Wurch T, & Colpaert FC (1996). Pharmacology of cloned human 5-HT1D receptor-mediated functional responses in stably transfected rat C6-glial cell lines: further evidence differentiating human 5-HT1D and 5-HT1B receptors. Naunyn-Schmiedeberg's Archives of Pharmacology 353: 144-156.

Pauwels PJ, Tardif S, Palmier C, Wurch T, & Colpaert FC (1997). How Efficacious are 5-HT1B/D Receptor Ligands: an Answer from GTPγS Binding Studies with Stably Transfected C6-glial Cell Lines. Neuropharmacology 36: 499-512.

Phebus LA, Johnson KW, Zgombick JM, Gilbert PJ, Van Belle K, Mancuso V, et al. (1997). Characterization of LY344864 as a pharmacological tool to study 5-HT1F receptors: Binding affinities, brain penetration and activity in the neurogenic dural inflammation model of migraine. Life Sciences 61: 2117-2126.

Razzaque Z, Heald MA, Pickard JD, Maskell L, Beer MS, Hill RG, et al. (1999). Vasoconstriction in human isolated middle meningeal arteries: determining the contribution of 5-HT(1B)- and 5-HT(1F)-receptor activation. British Journal of Clinical Pharmacology 47: 75-82.

Saxena PR, Vries PD, Wang W, Heiligers JPC, MaassenVanDenBrink A, Bax WA, et al. (1997). Effects of avitriptan, a new 5-HT1B/1D receptor agonist, in experimental models predictive of antimigraine activity and coronary side-effect potential. Naunyn-Schmiedeberg's Archives of Pharmacology 355: 295-302.

Schmuck K, Ullmer C, Kalkman HO, Probst A, & Lübbert H (1996). Activation of Meningeal 5-HT2B Receptors: An Early Step in the Generation of Migraine Headache? European Journal of Neuroscience 8: 959-967.

Shen Y, Monsma FJ, Metcalf MA, Jose PA, Hamblin MW, & Sibley DR (1993). Molecular cloning and expression of a 5-hydroxytryptamine7 serotonin receptor subtype. Journal of Biological Chemistry 268: 18200-18204.

Stanton JA, & Beer MS (1997). Characterisation of a cloned human 5-HT1A receptor cell line using [35S]GTPγS binding. European Journal of Pharmacology 320: 267-275.

Sternfeld F, Guiblin AR, Jelley RA, Matassa VG, Reeve AJ, Hunt PA, et al. (1999). Synthesis and Serotonergic Activity of 3-[2-(Pyrrolidin-1-yl)ethyl]indoles: Potent Agonists for the h5-HT1D Receptor with High Selectivity over the h5-HT1B Receptor. Journal of Medicinal Chemistry 42: 677-690.

Street LJ, Baker R, Davey WB, Guiblin AR, Jelley RA, Reeve AJ, et al. (1995). Synthesis and Serotonergic Activity of N,N-Dimethyl-2-[5-(1,2,4-triazol-1-ylmethyl)-1H-indol-3-yl]ethylamine and Analogs: Potent Agonists for 5-HT1D Receptors. Journal of Medicinal Chemistry 38: 1799-1810.

Vries PD, Villalón CM, & Saxena PR (1999). Pharmacology of triptans. Emerging Drugs 4: 107-125.

Wang C, Jiang Y, Ma J, Wu H, Wacker D, Katritch V, et al. (2013). Structural Basis for Molecular Recognition at Serotonin Receptors. Science 340: 610-614.

Wurch T, Palmier C, & Pauwels PJ (2000). Induction of a high-affinity ketanserin binding site at the 5-Hydroxytryptamine1B receptor by modification of its carboxy-terminal intracellular portion. Biochemical Pharmacology 59: 1117-1121.

Xu Y-C, Schaus JM, Walker C, Krushinski J, Adham N, Zgombick JM, et al. (1999). N-Methyl-5-tert-butyltryptamine: A Novel, Highly Potent 5-HT1D Receptor Agonist. Journal of Medicinal Chemistry 42: 526-531.

Zgombick JM, Schechter LE, Macchi M, Hartig PR, Branchek TA, & Weinshank RL (1992). Human gene S31 encodes the pharmacologically defined serotonin 5-hydroxytryptamine1E receptor. Molecular Pharmacology 42: 180-185.

Zgombick JM, Weinshank RL, Macchi M, Schechter LE, Branchek TA, & Hartig PR (1991). Expression and pharmacological characterization of a canine 5-hydroxytryptamine1D receptor subtype. Molecular Pharmacology 40: 1036-1042.

Zhang D, Kohlman D, Krushinski J, Liang S, Ying B-P, Reilly JE, et al. (2004). Design, synthesis and evaluation of bicyclic benzamides as novel 5-HT1F receptor agonists. Bioorganic & Medicinal Chemistry Letters 14: 6011-6016.
